# Golf Optimization Algorithm: A New Game-Based Metaheuristic Algorithm and Its Application to Energy Commitment Problem Considering Resilience

**DOI:** 10.3390/biomimetics8050386

**Published:** 2023-08-24

**Authors:** Zeinab Montazeri, Taher Niknam, Jamshid Aghaei, Om Parkash Malik, Mohammad Dehghani, Gaurav Dhiman

**Affiliations:** 1 Department of Electrical and Electronics Engineering, Shiraz University of Technology, Shiraz 7155713876, Iran; z.montazeri@sutech.ac.ir (Z.M.); m.dehghani@sutech.ac.ir (M.D.); 2 School of Engineering & Technology, Central Queensland University, Rockhampton 4701, Australia; j.aghaei@cqu.edu.au; 3 Department of Electrical and Software Engineering, University of Calgary, Calgary, AB T2N 1N4, Canada; maliko@ucalgary.ca; 4 Department of Electrical and Computer Engineering, Lebanese American University, Byblos 13-5053, Lebanon; gdhiman0001@gmail.com; 5 Department of Computer Science and Engineering, University Centre for Research and Development, Chandigarh University, Mohali 140413, India; 6 Department of Computer Science and Engineering, Graphic Era Deemed to be University, Dehradun 248002, India; 7 Division of Research and Development, Lovely Professional University, Phagwara 144411, India

**Keywords:** energy, energy carriers, exploitation, exploration, game-based, golf, metaheuristic algorithm, optimization, real-world applications, resilience

## Abstract

In this research article, we uphold the principles of the No Free Lunch theorem and employ it as a driving force to introduce an innovative game-based metaheuristic technique named Golf Optimization Algorithm (GOA). The GOA is meticulously structured with two distinctive phases, namely, exploration and exploitation, drawing inspiration from the strategic dynamics and player conduct observed in the sport of golf. Through comprehensive assessments encompassing fifty-two objective functions and four real-world engineering applications, the efficacy of the GOA is rigorously examined. The results of the optimization process reveal GOA’s exceptional proficiency in both exploration and exploitation strategies, effectively striking a harmonious equilibrium between the two. Comparative analyses against ten competing algorithms demonstrate a clear and statistically significant superiority of the GOA across a spectrum of performance metrics. Furthermore, the successful application of the GOA to the intricate energy commitment problem, considering network resilience, underscores its prowess in addressing complex engineering challenges. For the convenience of the research community, we provide the MATLAB implementation codes for the proposed GOA methodology, ensuring accessibility and facilitating further exploration.

## 1. Introduction

In tandem with the progressing frontiers of scientific and technological domains, an imperative arises for the development of more refined optimization methodologies capable of addressing the intricacies of novel as well as established optimization challenges. Metaheuristic algorithms, constituting stochastic-driven methodologies, offer a recourse for attaining acceptable solutions to optimization quandaries through their inherent capacity for random exploration of the solution space. These algorithms leverage stochastic operators and trial-and-error mechanisms to navigate the complex search domain [1]. However, it is important to note that the solutions yielded by metaheuristic algorithms do not carry an absolute assurance of attaining the global optimum [2]. Such solutions are aptly characterized as quasi-optimal solutions [3]. The underlying stochastic search process in metaheuristic algorithms operates on two distinct strata: the global level, encompassing exploration of the solution landscape, and the local level, which is centered around the concept of exploitation within localized regions. The pivotal determinant for the efficacy of metaheuristic algorithms in attaining viable solutions resides in the meticulous orchestration of a harmonious interplay between exploration and exploitation throughout the optimization endeavor [4]. The optimization process within metaheuristic algorithms follows an akin trajectory, commencing with the stochastic generation of a multitude of potential solutions, subsequently refined through iterative algorithmic cycles and the systematic application of diverse algorithmic steps. Upon the culmination of this iterative process, the most refined candidate solution is called the ultimate resolution to the given problem [5].

In the pursuit of enhanced and more apt solutions, a multitude of metaheuristic algorithms have been meticulously conceived and formulated by a diverse array of researchers. These algorithms have found extensive utilization across a myriad of scientific domains, encompassing realms such as energy systems [6,7], electrical engineering [8,9,10], predictive modeling [11], and energy management strategies [12,13,14], thereby manifesting their widespread applicability and utility.

In addition, with the evolution of technology and societal advancement, there has been an augmented demand for diverse forms of energy carriers. These encompass a spectrum of resources including oil, gas, coal, electricity, and renewable energy sources. The proficient utilization of these energy carriers not only facilitates the fulfillment of escalating energy requisites but also affords the opportunity for substantial operational cost savings [15]. Concurrently, power networks have remained inherently susceptible to an array of hazards, spanning from natural calamities to contemporary cyber threats. Consequently, the evaluation of the power system’s resilience against these potential disruptions assumes paramount importance. Resilience, in this context, denotes the capacity of the system to sustain an acceptable performance level in the face of severe disturbances and subsequently recover within a reasonable timeframe [16]. Optimal energy carrier operation during a specified study period hinges upon a meticulous consideration of energy consumption dynamics. The strategic utilization of energy carriers is meticulously determined, with the overarching objective of establishing a usage pattern that not only aligns with technical imperatives but also embodies the most economically judicious approach [17]. As such, the core aspiration of optimizing energy carrier operations is the identification of an optimal utilization pattern that prioritizes technical exigencies while concurrently optimizing economic considerations. One of the primary aims of this study revolves around the implementation of an energy commitment analysis within the energy network. This analysis, executed through the lens of optimization algorithms, is inherently attuned to resilience considerations, accentuating its significance within the broader research objectives.

At the heart of this inquiry lies a fundamental research question: in light of the myriad metaheuristic algorithms that have already been introduced, does the necessity persist for the introduction of novel metaheuristic approaches? The answer to this question, which is shaped by the tenets of the No Free Lunch (NFL) theorem [18], unfolds as follows: While a given metaheuristic algorithm may exhibit optimal performance when applied to a specific set of optimization problems, such success does not engender an unequivocal assurance of efficacy across all conceivable optimization scenarios. The NFL theorem underscores the absence of any presumptions, affirmative or negative, regarding an algorithm’s capacity to conquer a given optimization challenge. Consequently, it remains untenable to assert the supremacy of any singular metaheuristic algorithm as a universal optimizer for the entire spectrum of optimization applications. This theorem serves as a catalyst, urging researchers to continually forge ahead in their quest to devise superior solutions for optimization predicaments, thereby spurring the development of innovative metaheuristic algorithms.

The innovation and novelty of this paper are in the introduction and design of a new meta-heuristic algorithm called Golf Optimization Algorithm (GOA), which is used in the handling of optimization problems. The originality of the proposed approach is confirmed based on the best knowledge obtained from the literature review, where no metaheuristic algorithm inspired by the game of golf has been designed so far. Therefore, in this study, the Golf Optimization Algorithm (GOA) is designed and introduced for the first time in order to deal with optimization applications. The main contributions of this study are as follows:

- The fundamental inspiration of the GOA is to simulate the rules and behavior of players in the game of golf.
- Different phases of the GOA implementation in two phases of exploration and exploitation have been mathematically modeled.
- The efficiency of the GOA in solving optimization problems has been evaluated on fifty-two standard objective functions.
- The quality of the results obtained from GOA has been compared with ten well-known metaheuristic algorithms.
- The ability of the GOA to address real-world applications is tested on four engineering design problems and the optimization of operation of energy carriers’ problems with respect to energy grid resilience.

The rest of the article is as follows: In Section 2, the literature review is provided. The proposed GOA is introduced in Section 3. Simulation studies and results are presented in Section 4. The effectiveness of the GOA in solving the problem of the operation of energy carriers according to the resilience of the network is evaluated in Section 5. Finally, conclusions and several suggestions for future studies are provided in Section 6.

## 2. Literature Review

### 2.1. Metaheuristic Algorithms

Metaheuristic algorithms have been developed based on inspiration from various phenomena in nature, the natural behavior of living things, the laws of physics, biological sciences, human interactions, the rules of games, and other evolutionary processes. Based on the idea employed in the design, metaheuristic algorithms fall into nine groups: swarm-based, biology-based, physics-based, social-based, sports-based, music-based, chemistry-based, plant-based, and mathematics-based approaches. Also, based on the combination of these algorithms, hybrid approaches have also been developed [19].

Swarm-based approaches have been developed based on simulations of swarming activities of animals, birds, insects, aquatic animals, and other living organisms in nature. Particle Swarm Optimization (PSO) [20], Ant Colony Optimization (ACO) [21], Artificial Bee Colony (ABC) [22], and Firefly Algorithm (FA) [23] methods are among the most familiar swarm-based approaches. Moving the flock of birds and fish in search of food is the idea behind PSO. The ants’ attempt to identify the optimal path between the nest and the food source is the idea behind ACO. The activities of colony bees to gain access to food sources are the idea behind ABC. The behavior of fireflies and the flashing light pattern they produce based on the bioluminescence phenomenon is the idea behind FA. Coati Optimization Algorithm (COA) is developed based on simulating the strategy of coatis when attacking iguanas and when escaping from their predators [24]. Green Anaconda Optimization (GAO) is designed based on the mechanism of recognizing the position of the female species by the male species during the mating season and the hunting strategy of green anacondas [25]. Cat and Mouse-Based Optimizer (CMBO) is proposed based on simulation of movement of cats towards mice as well as the escape of mice towards havens [26]. Food search and hunting behavior have been the idea of designing different algorithms, such as Grey Wolf Optimizer (GWO) [27], Whale Optimization Algorithm (WOA) [28], African Vultures Optimization Algorithm (AVOA) [29], Marine Predator Algorithm (MPA) [30], Reptile Search Algorithm (RSA) [31], Tunicate Search Algorithm (TSA) [32], Orca Predation Algorithm (OPA) [33], White Shark Optimizer (WSO) [34], Pelican Optimization Algorithm (POA) [35], Serval Optimization Algorithm (SOA) [36], and Red Fox Optimization (RFO) [37].

Biology-based methods have been introduced based on the modeling of biological sciences and the concepts of natural selection. Genetic Algorithm (GA) [38] and Differential Evolution (DE) [39] are the most famous evolutionary algorithms that are developed based on modeling the reproductive process according to Darwin’s theory of evolution and the use of random operators such as selection, crossover, and mutation. Some other evolutionary-based algorithms are Evolutionary Programming (EP) [40], Genetic Programming (GP) [41], and Artificial Immune System (AIS) [42].

Physics-based algorithms are designed based on simulations of phenomena, processes, and laws of physics. Simulated Annealing (SA) [43] and Gravitational Search Algorithm (GSA) [44] are two well-known physics-based approaches. SA is inspired by the process of melting and cooling materials in metallurgy. In this physical process, the material is heated and slowly cooled under controlled conditions to increase the size of the crystal in the material and reduce its defects. GSA is inspired by the modeling of the gravitational force between different masses and the application of Newton’s laws of motion. Physical forces have been the source of inspiration in the design of algorithms such as Spring Search Algorithm (SSA) [45,46], which is based on spring force and Hook’s law, and Momentum Search Algorithm (MSA) [47], which is based on the simulation of the force resulting from the impact of objects colliding with each other. The physical phenomenon of rime-ice is employed in designing the RIME algorithm [48]. Some other physics-based algorithms are Multi-Verse Optimizer (MVO) [49], Fick’s Law Algorithm (FLA) [50], Equilibrium Optimizer (EO) [51], Kepler Optimization Algorithm (KOA) [52], Water Cycle Algorithm (WCA) [53], and Henry Gas Solubility Optimization (HGSO) [54].

Social-based approaches have been developed by modeling human activities and interactions in society. Interactions between students and the teacher in the classroom have been the idea behind Teaching–Learning Based Optimization (TLBO) [55]. Mother Optimization Algorithm (MOA) is introduced based on Eshrat’s care of her children in three phases education, advice, and upbringing [56]. Following the community leader to raise the level of development of that community has been the idea behind the Following Optimization Algorithm (FOA) [57]. The collaboration of individuals in a group to present teamwork and achieve team goals has been the idea behind Teamwork Optimization Algorithm (TOA) [58]. Learning different skills from instructors in schools has been the source of designing different algorithms, such as Driving Training-Based Optimization (DTBO) [59], Language Education Optimization (LEO) [60], Sewing Training-Based Optimization (STBO) [61], and Chef-Based Optimization Algorithm (CBOA) [62]. Some other human-based algorithms are Ali Baba and the Forty Thieves (AFT) [63], War Strategy Optimization (WSO) [64], Skill Optimization Algorithm (SOA) [65], Brain Storm Optimization (BSO) [66], and Poor and Rich Optimization (PRO) algorithm [67].

Sports-based metaheuristic algorithms are introduced based on simulating the rules and behavior of players in different individual or group games. Volleyball League simulation has been the source of inspiration behind the Volleyball Premier League (VPL) [68] and Football League simulation has been the source of inspiration behind the Football Game Based Optimization (FGBO) [15]. The players’ attempt to put the puzzle pieces together was the idea behind the Puzzle Optimization Algorithm (POA) [69]. The players’ effort to place the game rings in the score bars has been the main idea in the design of Ring Toss Game-Based Optimization (RTGBO) [70]. Some of the other game-based algorithms are Billiard Optimization Algorithm (BOA) [71], League Championship Algorithm (LCA) [72], Tug of War Optimization (TOA) [73], Running City Game Optimizer (RCGO) [74], and Soccer League Optimization (SLO) [75].

Music-based metaheuristic algorithms are developed inspired by musical concepts. Harmony Search (HS) [76], Melody Search (MS) [77], and Musical Composition Algorithm (MCA) [78] are examples of music-based algorithms. Chemical Reaction Optimization (CRO) [79] and Artificial Chemical Reaction Optimization Algorithm (ACROA) [80] are chemistry-based metaheuristic algorithms that are designed based on the simulation of chemical concepts. Plant-based metaheuristic algorithms are developed inspired by plant intelligence [81]. Some of the plant-based algorithms are Flower Pollination Algorithm (FPA) [82], Invasive Weed Optimization (IWO) [83], Paddy Field Algorithm (PFA) [84], Root Mass Optimization Algorithm (RMOA) [85], and Rooted Tree Optimization (RTO) [86]. Base Optimization Algorithm (BOA) [87], Subtraction–Average-Based Optimizer (SABO) [88], Selecting Some Variables to Update-Based Algorithm (SSVUBA) [89], Average and Subtraction-Based Optimizer (ASBO) [90], and Sine–Cosine Algorithm (SCA) [91] are examples of mathematics-based metaheuristic algorithms. Some other recently proposed metaheuristic algorithms are Monarch Butterfly Optimization (MBO) [92], Slime Mould Algorithm (SMA) [93], Moth Search Algorithm (MSA) [94], Hunger Games Search (HGS) [95], Runge Kutta method (RUN) [96], Colony Predation Algorithm (CPA) [97], weighted mean of vectors (INFO) [98], and Harris Hawks Optimization (HHO) [99].

Based on the best knowledge gained from the literature review, so far, golf game modeling has not been used in any metaheuristic algorithm. To address this, in this paper, a new metaheuristic algorithm based on golf simulation, which is described in the next section, is designed to address the optimization applications.

### 2.2. Energy Commitment

The intricate interplay between energy supply and demand has perpetually captured the attention of researchers, giving rise to a prolific body of scholarly investigations. A seminal milestone in this domain materialized through Charpentier’s seminal work in 1974, which constituted the inaugural comprehensive compilation of projects undertaken across diverse nations [59]. Within this seminal study, energy models are broadly categorized into two overarching classes: (1) single-fuel models, which encompass aspects such as energy carrier demand, production, distribution, and sector-specific optimization, and (2) multi-fuel models, which encompass the concurrent consideration of multiple energy carriers. The concept of energy commitment, which is fundamental to this discourse, pertains to the judicious operation of energy carriers. This entails, foremost, the fulfillment of energy demands and, subsequently, the alignment with economic considerations [100].

Underpinning the energy supply framework are a constellation of interdependent subsystems designed to furnish requisite energy to both economic and societal sectors. Diverse primary energy carriers, coupled with an array of processing and energy conversion technologies, collectively facilitate the realization of energy supply. The intricate flow of distinct energy forms constitutes an integral subset of the broader energy supply system. Notably, alterations in the flow of each energy carrier reverberate across the entirety of the energy supply system, intricately influencing the dynamics of other energy carriers’ flows [101,102].

Unit commitment (UC) has always been considered one of the main issues of power systems [103]. The main purpose and definition of UC is “to determine the most appropriate pattern of on or off power plants, so that first the technical issues are observed and then the most economical state”. Meeting the electrical energy demand with the least fuel cost with the optimal combination of different power plants is an important criterion in the UC problem [104]. Therefore, the basic constraint in the operation of power systems (regardless of grid losses) is the balance of total energy production of power plants with total electrical energy demand [105]. UC is a large-scale, nonlinear, and integer real-world optimization application that is quite challenging to solve. Considerable attention has been paid to optimization algorithms that solve UC over the past few decades. These techniques include comprehensive counting, priority listing, dynamic programming, integer and linear programming, Lagrange separation, fuzzy systems, artificial neural networks, taboo search, simulated annealing, genetic algorithms, and other metaheuristic algorithms [104].

Within the fabric of our modern infrastructure, industrial, commercial, and residential consumers are inextricably intertwined with a network of energy grids, encompassing diverse mediums such as electricity, natural gas, and localized heating or cooling systems. Notwithstanding the considerable scholarly focus on energy infrastructure, a noteworthy research gap persists in the exploration of the synergistic interplay between these distinct systems—a juncture that holds substantial untapped potential. This convergence offers an array of advantages, prominently featuring the amalgamation of complementary and adaptable attributes intrinsic to each system.

For instance, natural gas networks readily serve as cost-effective reservoirs, enabling straightforward energy storage. Conversely, electricity grids possess the intrinsic capacity to transmit energy over extensive distances, incurring relatively minimal losses. Therefore, the integration of these networks, capitalizing on their respective merits, stands poised to elevate system efficiency, bolster reliability, and optimize overall performance.

Numerous modeling paradigms have surfaced in the realm of multi-carrier energy systems. While prior models, as delineated in references [106,107], serve as rudimentary foundational constructs, they fall short of capturing the intricacies and flexibilities inherent to energy production and consumption cycles. In contrast, a more comprehensive and nuanced framework, expounded upon in [108], embraces the temporal and spatial interdependencies intrinsic to multi-objective optimization, thereby orchestrating the planning, design, and operation of multi-carrier energy systems.

A broader vista emerges within the realm of control-oriented modeling, as explored in [109], which lends itself to a more holistic understanding of multi-carrier energy systems. Integral to this orchestration are energy storage systems, which play an ancillary yet pivotal role. A novel reservoir-based storage approach, as documented in [110], delves into the assessment of storage performance and flexibility within the context of multi-carrier systems. Complementing this, a comprehensive inquiry into the reliability facets of multi-carrier energy systems is expounded upon in [110].

Within the overarching discourse, environmental considerations are not overlooked, as evidenced by the meticulous exploration of their impact, as elucidated in [111]. Thus, the research landscape continues to evolve, delving deeper into the intricate nexus of multi-carrier energy systems, unveiling novel vistas and innovative paradigms.

Maintaining the security of infrastructure systems such as power grids against natural disasters that occur with low impact and, of course, high probability has long been considered by the designers and operators of these systems. However, the operation of these systems is severely disrupted by severe disturbances. Therefore, it is necessary that, both in the design stage and in the operation stage of these systems, the behavior of the system in the event of severe accidents be studied and the necessary planning to address the shortcomings be considered. This behavior is known as a new feature called the resilience of an infrastructure system. Resilience refers to the temporal performance of a system including endurance, vulnerability, and reversibility in the event of a severe disturbance.

Therefore, the operation of the energy grid to meet energy demand with respect to grid resilience is an optimization challenge that must be solved using effective techniques. In the following sections, after designing the proposed optimization approach, the algorithm will be implemented on this optimization challenge.

## 3. Golf Optimization Algorithm

In this section, Golf Optimization Algorithm (GOA) theory is described, and then its mathematical modeling for use in optimization applications is presented.

### 3.1. Inspiration of GOA

Golf, an outdoor game or sport, unfolds on individual or team canvases, wielded by the adept manipulation of specialized clubs. The foundational tenets of this pastime dictate its essence—an artful journey of propelling a ball from its inaugural point towards a distant hole. This pursuit, executed through calculated swings and governed by a set of stipulations, encapsulates the essence of golf. Beneath this ostensibly straightforward surface, however, the game’s regulations interpose complexities, engendering a heightened level of challenge. Central to this enterprise is the strategic finesse required to guide the golf ball into the awaiting hole. This strategic choreography, a manifestation of intellectual prowess, serves as a wellspring of inspiration for the conceptualization of a pioneering metaheuristic algorithm. The inception of the Golf Optimization Algorithm (GOA) derives its blueprint from this very strategy, seamlessly weaving its contours into a methodological framework. In the realm of the GOA, this strategic dance finds embodiment, its intricate steps delineated, and its conceptual underpinnings crystallized through rigorous mathematical modeling.

### 3.2. Initialization of GOA

GOA is a population-based approach that can provide appropriate solutions to optimization problems through a random search of its members in the problem-solving space. The position of the GOA members in the problem search space determines the values of the problem variables. The population of GOA members can be mathematically represented using a matrix according to Equation (1). Like other metaheuristic algorithms, population members are randomly distributed over the problem space using a uniform distribution. The position of the GOA members at the beginning of the implementation of the algorithm is randomly initialized in the search space using Equation (2).

(1) $$X={\left[\begin{array}{c} X_{1}\\ \vdots \\ X_{i}\\ \vdots \\ X_{N} \end{array}\right]}_{N\times m}={\left[\begin{array}{ccccc} x_{1,1} & \cdots & x_{1,d} & \cdots & x_{1,m}\\ \vdots & \ddots & \vdots & ⋰ & \vdots \\ x_{i,1} & \cdots & x_{i,d} & \cdots & x_{i,m}\\ \vdots & ⋰ & \vdots & \ddots & \vdots \\ x_{N,1} & \cdots & x_{N,d} & \cdots & x_{N,m} \end{array}\right]}_{N\times m},$$

(2) $$X_{i}:x_{i,d}=lb_{d}+r\times (ub_{d}-lb_{d}),$$

Here, X is the population matrix of GOA, $X_{i}$ is the *i*th GOA member, $x_{i,d}$ is the value of the *d*th variable proposed by the *i*th GOA member, N is the number of GOA’s members, m is the number of variables, r is a random number in interval $\left[0-1\right]$, and $lb_{d}$ and $ub_{d}$ are the lower bound and upper bound of the *d*th variable, respectively.

Given the fact that each GOA member is a candidate solution to the problem and determines the problem variables, corresponding to each GOA member, a value for the objective function can be evaluated. The calculated values for the objective function can be represented using a vector according to Equation (3).

(3) $$F={\left[\begin{array}{c} F_{1}\\ \vdots \\ F_{i}\\ \vdots \\ F_{N} \end{array}\right]}_{N\times 1}={\left[\begin{array}{c} F(X_{1})\\ \vdots \\ F(X_{i})\\ \vdots \\ F(X_{N}) \end{array}\right]}_{N\times 1},$$

Here, F is the vector of objective function values and $F_{i}$ is the obtained value for objective function based on the *i*th GOA member.

Based on the comparison of the values obtained for the objective function, the member that provided the best value for the objective function is identified as the best member. Since the position of the GOA members and, consequently, the values of the objective function are updated in each iteration, the best member of the population must also be updated in each iteration.

### 3.3. Mathematical Model of GOA

After the initialization steps of the algorithm are completed, GOA enters the process of updating the population members. In GOA, members of the population are updated in two phases of exploration and exploitation.

#### 3.3.1. Phase 1: Exploration

The first swing in a game of golf is struck in an area of the playground called the grip. In the first swing, the players try to have the strongest shot towards the hole. In GOA, the position of the best member is considered the hole. This strategy scans different areas of the search space, indicating the exploration ability of the GOA in a global search. The process of updating GOA members based on the exploration phase is mathematically modeled using Equations (4) and (5). In this process, first, based on the simulation of the player’s strongest shot to the ball, a new position is calculated for each GOA member using Equation (4). Then, if the value of the objective function improves in this newly calculated position, it replaces the previous position of the corresponding member based on Equation (5). In the game of golf, players may hit shots where the ball passes the hole or approaches the hole. The use of parameter *I* in Equation (4) is to simulate this situation. If the parameter *I* is equal to 1, the ball approaches the hole. At the same time, in order to increase the exploration ability of the algorithm in the global search, if the parameter *I* is equal to 2, by increasing the possibility of moving the ball, the algorithm has more ability to scan different areas of the search space.

(4) $${X_{i}^{P1}:x}_{i,d}^{P1}=x_{i,d}+r\times (B_{d}-I\times x_{i,d})$$

(5) $$X_{i}=\left\{\begin{array}{c} X_{i}^{P1},\;F_{i}^{P1}<F_{i}\\ X_{i},\;else, \end{array}\right.$$

Here, $X_{i}^{P1}$ is the new calculated status of the *i*th GOA member based on the exploration phase, $x_{i,d}^{P1}$ is its *d*th dimension, $F_{i}^{P1}$ is its objective function value, B is the best member of GOA, $B_{d}$ is its *d*th dimension, r is a random number in interval $\left[0-1\right]$, and I is a random number that is selected randomly from the set of $\left\{1,2\right\}$.

#### 3.3.2. Phase 2: Exploitation

On the playground, the area where the hole is located is known as the green. In this area, players try to put the golf ball into the hole with kicks called putt. These precise kicks are provided with less power so that the golf ball does not move away from the green area and the hole. This strategy allows the area in which each GOA member is located to be carefully scanned, which indicates the exploitation ability of the GOA in a local search. The process of updating GOA members based on the exploitation phase is mathematically modeled using Equations (6) and (7). In this phase of the GOA update, a new position is calculated for each GOA member using Equation (6) based on the mathematical modeling of low-power shots of the player to the ball. This new position, if it improves the value of the objective function, replaces the previous position of the corresponding member according to Equation (7).

(6) $${X_{i}^{P2}:x}_{i,d}^{P2}=x_{i,d}+(1-2r)\times \frac{lb_{d}+r\times (ub_{d}-lb_{d})}{t}$$

(7) $$X_{i}=\left\{\begin{array}{c} X_{i}^{P2},\;F_{i}^{P2}<F_{i}\\ X_{i},\;else, \end{array}\right.$$

Here, $X_{i}^{P2}$ is the new calculated status of the *i*th GOA member based on exploitation phase, $x_{i,d}^{P2}$ is its *d*th dimension, $F_{i}^{P2}$ is its objective function value, and *t* is the iteration counter.

After each phase of updating the position of the population members, it should be checked whether the new solutions belong to the set of feasible solutions or not. The first group of constraints is related to the acceptable range for decision variables. If the value of any of the decision variables exceeds the upper or lower band, its value is set on the borderline values. This restriction of upper and lower bands for decision variables is checked and, if necessary, solved by using Equations (8) and (9).

(8) $$x_{i,d}^{P1}=\left\{\begin{array}{l} x_{i,d}^{P1},\;lb_{d}\leq x_{i,d}^{P1}\leq ub_{d}\\ ub_{d},\;x_{i,d}^{P1}>ub_{d}\\ lb_{d},\;x_{i,d}^{P1}<lb_{d}, \end{array}\right.$$

(9) $$x_{i,d}^{P2}=\left\{\begin{array}{l} x_{i,d}^{P2},\;lb_{d}\leq x_{i,d}^{P2}\leq ub_{d}\\ ub_{d},\;x_{i,d}^{P2}>ub_{d}\\ lb_{d},\;x_{i,d}^{P2}<lb_{d}, \end{array}\right.$$

The second group of constraints is related to the equal and unequal constraints of the optimization problem. In order to deal with these limitations, the penalty factor has been used. If any of the equal or unequal constraints are not met, it means that the new solution does not belong to the set of feasible solutions. Therefore, by adding the penalty coefficient to the value of the objective function of the problem, the new solution is recognized as an inappropriate solution, and it will not be possible to choose that solution as the solution to the problem. This group of constraints has been checked using Equation (10).

(10) $$F_{i}=F_{i}+n_{q}\times PF_{i}$$

Here, $n_{q}$ is the number of constraints of the problem that are not established and $PF_{i}$ is the penalty factor in which $PF_{i}=10^{5}\times \left|F_{i}\right|$.

### 3.4. Repetition Process, Pseudocode, and Flowchart of GOA

**Figure 1:**
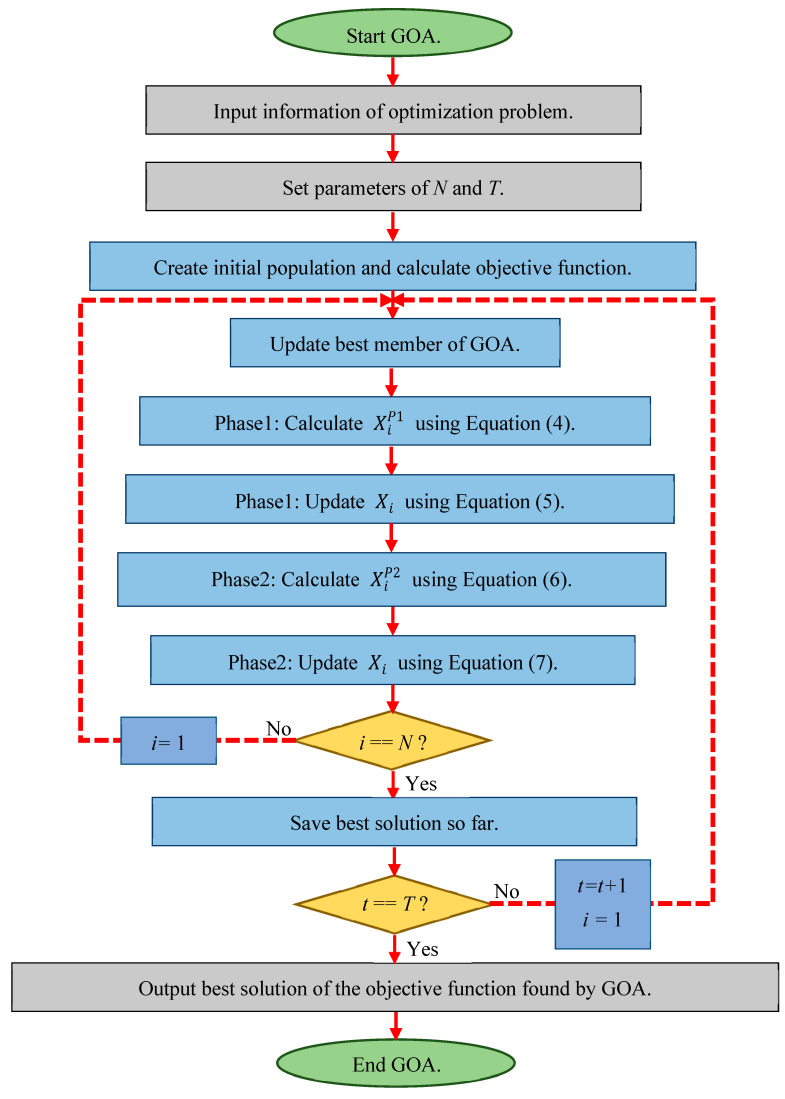
Flowchart of the GOA.

The first iteration of the GOA is completed after updating all its members based on the first and second phases. Based on the new values obtained, the GOA enters the next iteration, and the update process is repeated based on Equations (4)–(10) until the algorithm is fully implemented. Finally, the best candidate solution discovered during the iteration of the algorithm is introduced as the solution to the problem. The flowchart of GOA implementation steps is presented in Figure 1 and its pseudocode is presented in Algorithm 1. The complete set of codes is available at the following repository: https://www.mathworks.com/matlabcentral/fileexchange/133817-golf-optimization-algorithm-goa. (accessed on 13 August 2023)
**Algorithm 1.** Pseudocode of the GOA.Start GOA.1.Input the optimization problem information.2.Set *T* (number of iterations) and *N* (number of GOA members).3.For *t* = 1:*T*4.Update best member of GOA as hole.5. For *i* = 1:*N*7.  Phase 1:8.  Calculate new status of *i*th GOA member based on exploration phase of GOA using Equation (4).9.  Update *i*th GOA member using Equation (5).10.  Phase2: Exploitation11.  Calculate new status of *i*th GOA member based on exploitation phase of GOA using Equation (6).12.  Update *i*th GOA member using Equation (7).13. end14. Save best candidate solution so far.15.end16.Output best obtained solution.End GOA.

### 3.5. Computational Complexity

In this subsection, the computational complexity of the GOA is analyzed. The initialization of the GOA for a problem with the number *m* of the decision variable is equal to *O*(*Nm*), where *N* is the number of GOA members. In each iteration, the GOA member update process is performed in two phases, which have a computational complexity equal to *O*(2*NmT*), where *T* is the maximum number of iterations of the algorithm. Accordingly, the total computational complexity of the GOA is equal to *O*(*Nm* (1 + 2*T*)).

## 4. Simulation Studies and Results

In this section, the capability of the GOA in handling optimization problems and providing solutions is evaluated. For this purpose, a set of fifty-two standard objective functions of unimodal and multimodal types [112], as well as the CEC 2017 test suite, are employed. To analyze the ability of the GOA in optimization applications, its results have been compared with ten metaheuristic algorithms: GA, PSO, GSA, TLBO, MVO, GWO, WOA, MPA, TSA, and RSA. From the numerous metaheuristic algorithms designed so far, ten algorithms have been selected for comparison with the proposed GOA algorithm. The reason for choosing these ten competitor algorithms is that GA and PSO are the best-known and most widely used metaheuristic algorithms. GSA, TLBO, GWO, MVO, and WOA, introduced between 2009 and 2016, have been popular methods for researchers and have been widely cited. MPA, TSA, and RSA are recently published metaheuristic algorithms that have quickly gained the attention of scientists and have been used in a variety of real-world applications. The control parameters are adjusted as specified in Table 1. Regarding competitor algorithms, GA, PSO, GSA, GWO, MVO, WOA, MPA, TSA, and RSA have a time complexity equal to O(Nm (1+T)), and TLBO has a computational complexity equal to O(Nm (1+2T)). Therefore, it is clear that the proposed GOA approach has higher computational complexity than GA, PSO, GSA, GWO, MVO, WOA, MPA, TSA, and RSA as well as similar computational complexity to TLBO. However, to make a fair comparison, we used the population size of each metaheuristic algorithm in the simulation analysis so that the total number of function evaluations is the same for all employed algorithms. The population size for GOA and TLBO is considered equal to 30 members, and for GA, PSO, GSA, GWO, MVO, WOA, MPA, TSA, and RSA it is considered equal to 60 members. The proposed GOA approach and competitor algorithms are each implemented in twenty independent runs in optimizing each objective function. The proposed GOA approach and competitor algorithms are each implemented in twenty independent runs in optimizing each objective function. To optimize functions F1 to F23, GOA and each competitor algorithm are used in twenty independent runs with 50,000 function evaluations (i.e., FEs=50,000). For solving the CEC 2017 test suite, the proposed GOA and the competitor algorithms are employed in fifty-one independent runs, each containing 110,000∙m function evaluations (i.e., FEs=10,000∙m), where m is the number of problem variables set to 10. Simulation results are reported using six indicators: mean, best, worst, standard deviation (std), median, and rank. Experiments have been implemented on the software MATLAB R2022a using a 64-bit Core i7 processor with 3.20 GHz and 16 GB main memory.

### 4.1. Evaluation of Unimodal Functions

The selected unimodal objective functions, including F1 to F7, have no local optimum, and the purpose of their optimization is to evaluate the exploitation ability of optimization algorithms. The implementation results of the GOA and competitor algorithms on F1 to F7 are reported in Table 2. Based on the obtained results, the proposed algorithm with high exploitation ability has provided the global optimal solution for functions F1, F2, F3, F4, F5, and F6. In addition, in F7 optimization, the proposed algorithm is the first best optimizer compared to competitor algorithms. The analysis of the simulation results shows that the proposed GOA has provided far superior results compared to the competitor algorithms and has a high exploitation ability compared to the competitor algorithms.

### 4.2. Evaluation of High-Dimensional Multimodal Functions

The selected high-dimensional multimodal objective functions, including F8 to F13, are complex problems that, in addition to the global optimum, have many local optimums. Therefore, these functions are suitable for analyzing the exploration ability of optimization algorithms in the global search of the problem-solving space. The optimization results of F8 to F13 functions using GOA and competitor algorithms are reported in Table 3. Based on the results, the proposed algorithm with its high exploration ability has well identified the main optimal region for functions F9 and F11 and converged to the global optimum. Moreover, the proposed approach is the first best optimizer for functions F8, F10, F12, and F13. The analysis of the simulation results indicates that the proposed algorithm, with its high exploration ability, has provided a very superior performance compared to the competitor algorithms in handling F8 to F13 high-dimensional multimodal objective functions.

### 4.3. Evaluation of Fixed-Dimensional Multimodal Functions

**Figure 2:**
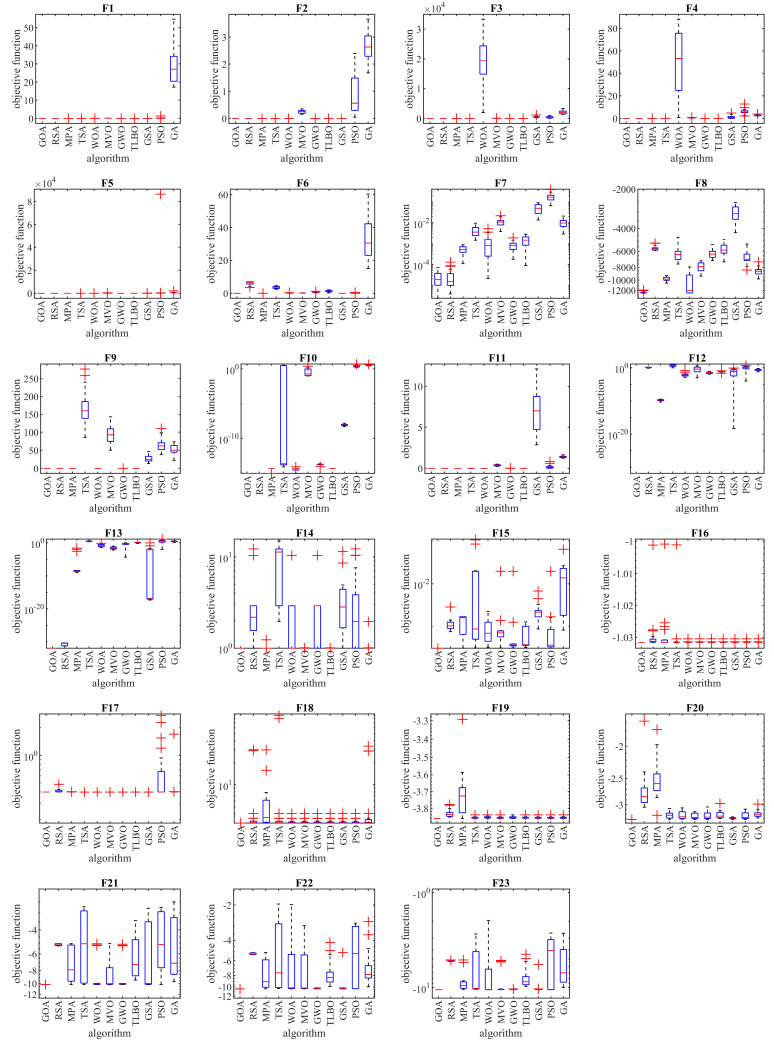
Boxplots of the GOA and competitor algorithms on F1 to F23 test functions.

**Figure 3:**
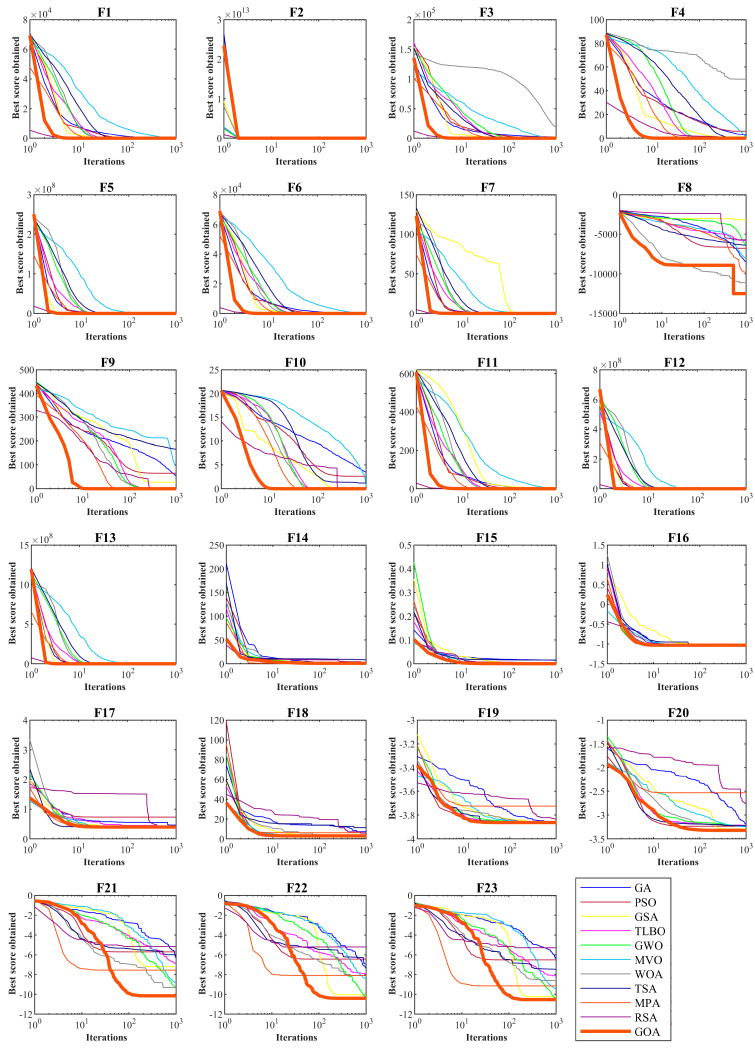
Convergence curves of the GOA and competitor algorithms on F1 to F23 test functions.

The selected fixed-dimensional multimodal objective functions, including F14 to F23, in addition to the global optimum, have a limited number of local optimums. This feature makes these functions suitable for analyzing the ability of metaheuristic algorithms to balance exploration and exploitation. The optimization results of functions F14 to F23 are reported in Table 4. The simulation results show that the proposed GOA is the first best optimizer for function F15. In addition, in other cases where the GOA has similar conditions in the mean criterion with other competitor algorithms, the proposed GOA has performed better by providing better values for the std index. The analysis of the simulation results shows that the proposed GOA has performed better compared to competitor algorithms in optimizing functions F14 to F23 and has a superior ability in creating a balance between exploration and exploitation compared to competitor algorithms. Boxplot diagrams and convergence curves of metaheuristic algorithms and GOA in handling functions F1 to F23 are drawn in Figure 2 and Figure 3.

Based on the obtained results, it can be seen that the proposed GOA approach has provided better or similar solutions to some competing algorithms in most benchmark functions F1 to F23. The question that is raised is as follows: is this superiority of the GOA not in contrast with the concept of the NFL theorem? In response to this question, it should be explained that according to the NFL theorem, the superior performance of the GOA in solving a set of benchmark functions is no guarantee for the same performance of the proposed approach in solving other optimization applications. It should also be noted that functions F1 to F23 are only a small set of the world of optimization problems. Therefore, in solving other optimization problems, the GOA may not provide better performance compared to competing algorithms. As seen in the next subsection, in handling some benchmark functions from the CEC 2017 test suite, the proposed GOA approach was not able to provide better results compared to competing algorithms. On the other hand, there is always the possibility that newer metaheuristic algorithms will be designed and developed that have better performance compared to the GOA. Therefore, it is by no means claimed that the GOA is the best optimizer for all optimization applications. According to this, the results obtained from GOA’s performance have no contrast with the NFL theorem.

### 4.4. Evaluation of the CEC 2017 Test Suite

**Figure 4:**
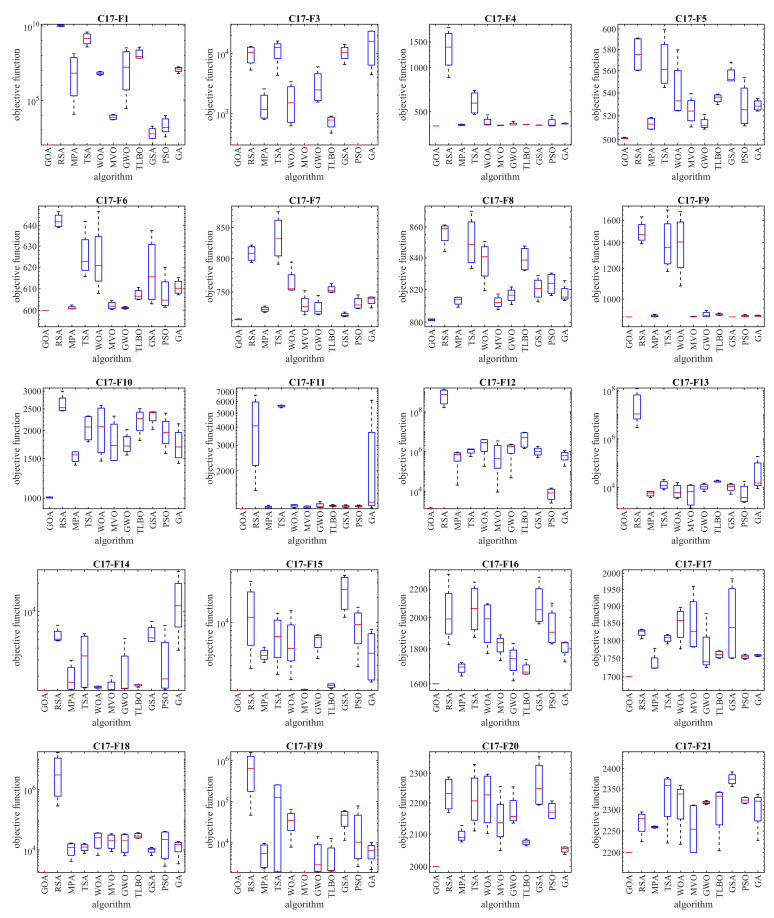

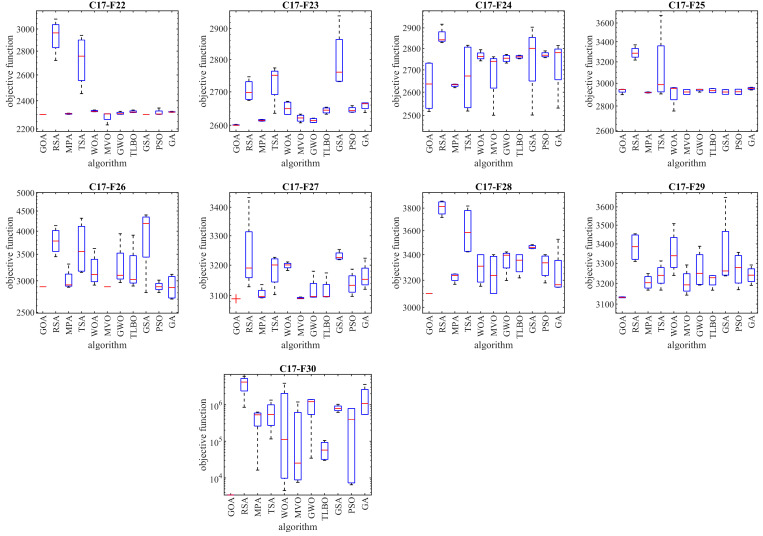
Boxplots of the GOA and competitor algorithms on the CEC 2017 test suite.

In this subsection, the efficiency of the GOA in handling the CEC 2017 test suite is evaluated. This test suite has thirty benchmark functions consisting of three unimodal functions of C17-F1 to C17-F3, seven multimodal functions of C17-F4 to C17-F10, ten hybrid functions of C17-F11 to C17-F20, and ten composition functions of C17-F21 to C17-F30. Full details and a description of the CEC 2017 test suite are provided in [113]. The implementation results of the GOA and competitor algorithms on the CEC 2017 test suite are reported in Table 5. The boxplot diagrams obtained from the performance of metaheuristic algorithms are drawn in Figure 4. Based on the obtained results, GOA is the first best optimizer for functions C17-F1, C17-F3 to C17-F21, C17-F23, C17-F24, and C17-F27 to C17-F30. The analysis of the optimization results shows that the proposed GOA approach has provided better results in most of the benchmark functions; while obtaining the first rank of the best optimizer, it has provided superior performance in handling the CEC 2017 test suite compared to competitor algorithms.

### 4.5. GOA for Real-world Applications

In this subsection, the performance of GOA in handling real-world applications is challenged. For this purpose, GOA and competing algorithms have been implemented on four engineering design problems named tension/compression spring (TCS) design, welded beam (WB) design, speed reducer (SR) design, and pressure vessel (PV) design. The mathematical model and full description of these real-world applications are provided for TCS in [28], for WB in [28], for SR in [114,115], and for PV in [116]. The mathematical model of these real-world applications is as follows:

- Mathematical model of TCS:
  Consider $X=\left[x_{1},x_{2},x_{3}\right]=\left[d,D,P\right].$
  Minimize $f\left(x\right)=\left(x_{3}+2\right)x_{2}x_{1}^{2}.$
  Subject to
  $$\begin{array}{l} g_{1}\;\left(x\right)=1-\frac{x_{2}^{3}x_{3}}{71,785x_{1}^{4}}\;\leq \;0,\\ g_{2}\;\left(x\right)=\frac{4x_{2}^{2}-x_{1}x_{2}}{12,566(x_{2}x_{1}^{3})}+\frac{1}{5108x_{1}^{2}}-1\leq \;0,\\ g_{3}\;\left(x\right)=1-\frac{140.45x_{1}}{x_{2}^{2}x_{3}}\leq \;0,\\ g_{4}\;\left(x\right)=\frac{x_{1}+x_{2}}{1.5}-1\;\leq \;0. \end{array}$$
  With
  $$0.05\leq x_{1}\leq 2,\;{0.25\leq x}_{2}\leq 1.3\;and\;2\leq \;x_{3}\leq 15.$$
- Mathematical model of WB:
  Consider $X=\left[x_{1},\;x_{2},\;x_{3},\;x_{4}\right]=\left[h,\;l,\;t,\;b\right]$.
  Minimize $f\;(x)=1.10471x_{1}^{2}x_{2}+0.04811x_{3}x_{4}\;(14.0+x_{2})$.
  Subject to
  $$\begin{array}{l} g_{1}\;\left(x\right)=\tau \left(x\right)-13,600\;\leq \;0,\\ g_{2}\;\left(x\right)=\sigma \left(x\right)-30,000\;\leq \;0,\\ g_{3}\;\left(x\right)=x_{1}-x_{4}\leq \;0,\\ g_{4}\;(x)=0.10471x_{1}^{2}+0.04811x_{3}x_{4}\;(14+x_{2})-5.0\;\leq \;0,\\ g_{5}(x)=0.125-x_{1}\leq \;0,\\ g_{6}(x)=\delta \;(x)-0.25\;\leq \;0,\\ g_{7}\;(x)=6000-p_{c}\;(x)\;\leq \;0, \end{array}$$
  where
  $$\begin{array}{l} \tau \left(x\right)=\sqrt{\tau^{\prime }+\left(2\tau \tau^{\prime }\right)\frac{x_{2}}{2R}+{\left(\tau^{\prime \prime }\right)}^{2}},\\ \tau^{\prime }=\frac{6000}{\sqrt{2}x_{1}x_{2}},\\ \tau^{\prime \prime }=\frac{MR}{J},\\ M=6000\left(14+\frac{x_{2}}{2}\right),\\ R=\sqrt{\frac{x_{2}^{2}}{4}+{\left(\frac{x_{1}+x_{3}}{2}\right)}^{2}},\\ J=2\left\{x_{1}x_{2}\sqrt{2}\left[\frac{x_{2}^{2}}{12}+{\left(\frac{x_{1}+x_{3}}{2}\right)}^{2}\right]\right\},\\ \sigma \left(x\right)=\frac{504,000}{x_{4}x_{3}^{2}}\\ \delta \;\left(x\right)=\frac{65,856,000}{\left(30∙10^{6}\right)x_{4}x_{3}^{3}},\\ p_{c}\;\left(x\right)=\frac{4.013\left(30∙10^{6}\right)\sqrt{\frac{x_{3}^{2}x_{4}^{6}}{36}}}{196}\left(1-\frac{x_{3}}{28}\sqrt{\frac{30∙10^{6}}{4(12∙10^{6})}}\right). \end{array}$$
  With
  $$0.1\leq x_{1},\;x_{4}\leq 2\;and\;0.1\leq x_{2},\;x_{3}\leq 10.$$
- Mathematical model of SR:
  Consider $X=\left[x_{1,}\;x_{2},\;x_{3},\;x_{4},\;x_{5}{\;,x}_{6}\;,x_{7}\right]=\left[b,\;m,\;p,\;l_{1},\;l_{2},\;d_{1},\;d_{2}\right]$.
  Minimize $f\;\left(x\right)=0.7854x_{1}x_{2}^{2}\left(3.3333x_{3}^{2}+14.9334x_{3}-43.0934\right)-1.508x_{1}\left(x_{6}^{2}+x_{7}^{2}\right)+7.4777\left(x_{6}^{3}+x_{7}^{3}\right)+0.7854(x_{4}x_{6}^{2}+x_{5}x_{7}^{2})$.
  Subject to
  $$\begin{array}{l} g_{1}\;\left(x\right)=\frac{27}{x_{1}x_{2}^{2}x_{3}}-1\;\leq \;0,\\ g_{2}\;\left(x\right)=\frac{397.5}{x_{1}x_{2}^{2}x_{3}}-1\leq \;0,\\ g_{3}\;\left(x\right)=\frac{1.93x_{4}^{3}}{x_{2}x_{3}x_{6}^{4}}-1\leq \;0,\\ g_{4}\;\left(x\right)=\frac{1.93x_{5}^{3}}{x_{2}x_{3}x_{7}^{4}}-1\;\leq \;0,\\ g_{5}\left(x\right)=\frac{1}{110x_{6}^{3}}\sqrt{{\left(\frac{745x_{4}}{x_{2}x_{3}}\right)}^{2}+16.9\times 10^{6}}-1\leq \;0,\\ g_{6}(x)=\frac{1}{85x_{7}^{3}}\sqrt{{\left(\frac{745x_{5}}{x_{2}x_{3}}\right)}^{2}+157.5\times 10^{6}}-1\;\leq \;0,\\ g_{7}\;\left(x\right)=\frac{x_{2}x_{3}}{40}-1\;\leq \;0,\\ g_{8}\;\left(x\right)=\frac{{5x}_{2}}{x_{1}}-1\;\leq \;0,\\ g_{9}\;\left(x\right)=\frac{x_{1}}{12x_{2}}-1\;\leq \;0,\\ g_{10}\;\left(x\right)=\frac{{1.5x}_{6}+1.9}{x_{4}}-1\;\leq \;0,\\ g_{11}\;\left(x\right)=\frac{{1.1x}_{7}+1.9}{x_{5}}-1\;\leq \;0. \end{array}$$
  With
  $$2.6\leq x_{1}\leq 3.6,\;0.7\leq x_{2}\leq 0.8,\;17\leq x_{3}\leq 28,\;7.3\leq x_{4}\leq 8.3,\;7.8\leq x_{5}\leq 8.3,\;2.9\leq x_{6}\leq 3.9,\;and\;5\leq x_{7}\leq 5.5\;.$$
- Mathematical model of PV:
  Consider $X=\left[x_{1},\;x_{2},\;x_{3},\;x_{4}\right]=\left[T_{s},\;T_{h},\;R,\;L\right]$.
  Minimize $f\;\left(x\right)=0.6224x_{1}x_{3}x_{4}+1.778x_{2}x_{3}^{2}+3.1661x_{1}^{2}x_{4}+19.84x_{1}^{2}x_{3}.$
  Subject to
  $$\begin{array}{l} g_{1}\;\left(x\right)=-x_{1}+0.0193x_{3}\;\leq \;0,\\ g_{2}\;\left(x\right)=-x_{2}+0.00954x_{3}\leq \;0,\\ g_{3}\;\left(x\right)=-\pi x_{3}^{2}x_{4}-\frac{4}{3}\pi x_{3}^{3}+1,296,000\leq \;0,\\ g_{4}\;\left(x\right)=x_{4}-240\;\leq \;0. \end{array}$$
  With
  $$0\leq x_{1},x_{2}\leq 100,\;{and\;10\leq x}_{3},x_{4}\leq 200.$$

**Figure 5:**
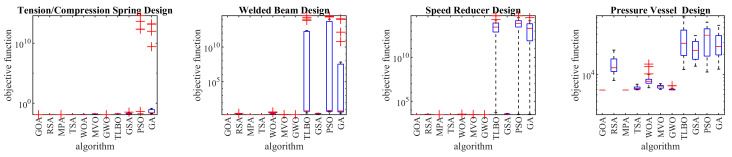
Boxplot diagrams of metaheuristic algorithms on real-world applications.

The optimization results of these engineering challenges are reported in Table 6. The boxplot diagrams obtained from the performance of metaheuristic algorithms are drawn in Figure 5. The analysis of the optimization results shows that the proposed GOA approach has provided the optimal design for the TCS problem with the corresponding objective function value equal to 0.012665. In dealing with the WB problem, the proposed GOA approach has provided the optimal design with the corresponding objective function value equal to 1.724852. GOA has provided the optimal design for the SR problem with the corresponding objective function value equal to 2996.348. In dealing with the PV problem, the proposed GOA approach with the corresponding objective function value equal to 5882.901 has provided the optimal design. The analysis of optimization results shows that GOA is effective in dealing with real-world applications, and compared to competitor algorithms, it has provided superior performance by providing better results.

### 4.6. Statistical Analysis

In this subsection, statistical analysis was used to check whether or not the superiority of the proposed GOA approach compared to competing algorithms is significant from a statistical point of view. To answer this question, the Wilcoxon rank sum test [117] was employed, which is a non-parametric test and is used to determine the significant difference between the average of two data samples. In this test, using the *p*-value index, it is determined whether or not there is a significant difference between the performance of the two algorithms.

The results of implementing the Wilcoxon rank sum test on the performance of the GOA and competitor algorithms are reported in Table 7. Based on the results, in cases where the *p*-value is less than 0.05, the proposed GOA approach has a significant statistical superiority in comparison with the corresponding competitor algorithm. Therefore, it is evident that GOA has significant statistically superior performance in handling all benchmarks and engineering problems compared to competitor algorithms.

### 4.7. Discussion

Metaheuristic algorithms are stochastic techniques that are able to achieve suitable solutions for optimization problems based on a random search in the problem-solving space in an iterative-based process. There are three important principles in the optimization process using metaheuristic algorithms: exploration, exploitation, and balancing during the search process.

Because unimodal functions do not have any local optimum, they are suitable options for evaluating the exploitation power of metaheuristic algorithms in a local search and converging towards the global optimum. Functions F1 to F7, as well as C17-F1 and C17-F3 from the CEC 2017 test suite, are unimodal functions. Based on the simulation results, GOA has been able to exactly converge to the global optimum in the optimization of functions F1, F2, F3, F4, F5, F6, C17-F1, and C17-F3 with high ability in exploitation and provide powerful local search. Also, in solving the F7 function, GOA is the first best optimizer compared to competing algorithms. Based on the simulation results, it is evident that the proposed GOA approach has a high power in exploitation in order to manage an effective local search in the problem-solving space. Overall, GOA has provided superior performance in the optimization of unimodal functions by obtaining the rank of the first best optimizer compared to competing algorithms. In addition, the statistical analysis shows that the superiority of the proposed GOA approach in handling unimodal benchmark functions compared to competing algorithms is significant from a statistical point of view.

High-dimensional multimodal functions have several local optima in the search space in addition to global optima. This feature makes it a challenge for metaheuristic algorithms to achieve the main global optimum among local extrema. High-dimensional multimodal functions are suitable options in order to evaluate the exploration power of metaheuristic algorithms in providing the global search of the problem-solving space with the aim of discovering the area containing the global optimum. In this regard, with the aim of measuring the exploration power of the GOA and competing metaheuristic algorithms, functions F8 to F13 and C17-F4 to C17-F10 have been selected from the high-dimensional multimodal type. Based on the simulation results, GOA has been able to exactly achieve the global optimum in solving functions F9, F11, C17-F4, C17-F6, and C17-F9, with high power in exploration and global search. Also, in solving functions F8, F10, F12, F13, C17-F5, C17-F7, C17-F8, and C17-F10, the proposed GOA approach is the first best optimizer. What can be concluded from the simulation results is that the proposed GOA approach has a high power in exploration and global search and is able to identify the area containing the main optimum in the problem-solving space. In general, GOA has provided superior performance compared to competing algorithms by providing better results in most high-dimensional multimodal functions and obtaining the rank of the first best optimizer. Statistical analysis also shows that GOA has a significant statistical superiority in solving high-dimensional multimodal functions compared to competing algorithms.

Fixed-dimensional multimodal functions have fewer local optima compared to high-dimensional multimodal functions. These functions are suitable options in order to evaluate the ability of metaheuristic algorithms in balancing exploration and exploitation during the search process. For this purpose, the functions F14 to F23 have been selected from the fixed-dimensional multimodal functions. Also, the functions C17-F11 to C17-F30 are complex optimization problems that challenge the ability of metaheuristic algorithms to establish a balance between exploration and exploitation. Based on the simulation results, GOA with high ability in balancing exploration and exploitation has been able to identify the region containing the main optimum and converge towards solutions close to the global optimum. The comparison of the simulation results shows that GOA is the first best optimizer for functions F14 to F23, C17-F11 to C17-F21, C17-F23, C17-F24, and C17-F27 to C17-F30 compared to competing algorithms. What is evident from the simulation results is the GOA has provided superior performance in solving these functions with a better ability to balance exploration and exploitation compared to competing algorithms. Citing the results of statistical analysis also shows that GOA has a significant statistical superiority against competing metaheuristic algorithms.

One of the most important applications of metaheuristic algorithms is their employment in handling real-world applications. In fact, it should be checked whether or not the use of metaheuristic algorithms in optimizing real-world applications can be fruitful. In this regard, four engineering design problems have been selected from real-world applications. The optimization results show that GOA has provided superior performance compared to competing algorithms by providing optimal design for all four engineering problems. Analysis of the optimization results shows that GOA has an effective and acceptable performance in handling real-world applications.

## 5. Energy Commitment Problem and Resilience

In this section, to evaluate the performance of the proposed approach in real-world applications, GOA’s ability to address the engineering problem of the operation of energy carriers with respect to energy network resilience is challenged.

### 5.1. Case Study

The operation of energy carriers using the proposed GOA approach is studied on an energy network with twenty-six power plant units. The complete information of this network, the mathematical formulation, and the objective function of this problem are provided in reference [118]. The main goal of this optimization problem is to reduce the cost of energy carriers’ operation in order to meet the energy demand. The objective function and constraints of the energy commitment problem are as follows:

(11) $$F_{objective}=min\left\{\sum_{t=1}^{T}\left[\sum_{i=1}^{N_{c}}carrier_{i}^{t}\times price_{i}+\sum_{i=1}^{N_{g}}SC_{i}^{t}+\sum_{i=1}^{N_{g}}C_{i}u_{i}^{t}\right]\;\right\}$$

(12) $$SC_{i}^{t}=\left\{\begin{array}{c} SC_{i},\;u_{i}^{t}>u_{i}^{t-1}\\ 0,\;else \end{array}\right.$$

(13) $$P_{g_{i}}^{min}\leq P_{g_{i}}\leq P_{g_{i}}^{max}$$

(14) $$\sum_{i=1}^{N_{g}}P_{g_{i}}^{t}=load^{t}$$

Here, $F_{objective}$ is the objective function of the EC problem, *T* is the study period, $N_{c}$ is the number of various carriers, $carrier_{i}^{t}$ is the need of *i*-th carrier in the *t*-th hour and $price_{i}$ is its price, $N_{g}$ is the number of units, $SC_{i}^{t}$ is the start-up cost for the *i*-th unit in the *t*-th hour, $C_{i}$ is the fixed cost for the *i*-th unit, $u_{i}^{t}$ is the status (on or off) of it in the *t*-th hour, $P_{g_{i}}$ is the production of the *i-*th unit and $load^{t}$ is the electricity demand in the *t*-th hour.

This study is carried out in two normal and abnormal situations, where one of the power plants is out of the network based on natural events, and the resilience of the network against this event should be analyzed.

### 5.2. Operation of the Energy Network in Normal Mode

The implementation results of the GOA and competitor algorithms on the mentioned problem are presented in Table 8. Simulation results show that GOA has good performance in optimizing the operation of energy carriers’ problem, and compared to competitor algorithms, it is the first best optimizer for this problem.

### 5.3. Operation of the Energy Network in Abnormal Mode Considering Resilience

In the subsequent investigation, a hypothetical scenario is postulated where, driven by the impact of natural calamities, a critical event unfolds during the network’s operation. Specifically, in the 20th hour of the designated study period, the 12th power plant encounters a catastrophic incident, resulting in the incapacitation of its capacity to generate electrical energy. Consequently, this predicament necessitates a meticulous assessment of the energy network’s resilience. The overarching objective revolves around ascertaining whether the network’s operational integrity would falter under these exigent circumstances or if it would successfully sustain the provisioning of requisite energy.

The outcomes stemming from the implementation of the Golf Optimization Algorithm (GOA) and its competitor algorithms within the context of this accident-induced adversity are comprehensively delineated in Table 9. The ascertained results manifestly underscore GOA’s prominence as the preeminent optimizer in navigating the intricate labyrinth of the energy commitment quandary, especially when contrasted against its algorithmic counterparts. Delving into the intricacies of the simulation findings for both normal and abnormal operational paradigms reveal a conspicuous trend: the introduction of the catastrophic incident, while increasing the operational expenditure of the network, does not undermine its overarching resilience. In essence, the network steadfastly maintains its capacity to withstand and endure the disruptive influence of the natural disaster, thereby affirming its steadfast resilience.

## 6. Conclusions and Future Works

This research paper introduced a novel game-based metaheuristic algorithm named the Golf Optimization Algorithm (GOA). The conceptual foundation of the GOA stems from an emulation of the regulations governing the game of golf, coupled with strategic considerations reflective of players’ tactics within the game. The procedural intricacies of the GOA were elaborated upon, followed by a mathematical representation demarcating its dual-phase operation: an exploratory phase dedicated to global search and an exploitative phase tailored for local search. The evaluative prowess of the GOA in tackling optimization quandaries was scrutinized across a comprehensive spectrum encompassing fifty-two standard benchmark functions, spanning both unimodal and multimodal typologies, as well as the CEC 2017 test suite. Evidential from optimization outcomes is GOA’s adeptness in concurrently preserving a symbiotic equilibrium between explorative and exploitative pursuits, resulting in the identification of primary optima and efficient global convergence.

In comparative light, this study juxtaposed GOA’s performance against ten preeminent metaheuristic counterparts, divulging GOA’s superior and markedly competitive standing. Furthermore, the practical applicability of the GOA was demonstrated through its adept navigation of four real-world engineering design challenges. In particular, its deployment in optimizing energy carrier operations vis-à-vis grid resilience substantiates GOA’s efficacy in confronting real-world scenarios.

GOA accrues various merits, especially its parsimonious parameterization necessitating only two common parameters, N (population size) and T (maximum iteration count). This attribute precludes the need for a meticulous parameter tuning procedure. The algorithm’s approachability, facile implementation, and underlying conceptual clarity serve as supplementary advantageous attributes. The ability of the GOA to strike a judicious harmony between exploratory and exploitative tendencies during the search process in problem solution spaces underscores another commendable facet.

However, it is imperative to acknowledge GOA’s inherent limitations. As a member of the stochastic algorithm category, it remains susceptible to the absence of a deterministic guarantee in achieving global optimality, constituting an inherent drawback. Additionally, adhering to the No Free Lunch (NFL) theorem, the algorithm does not assert universal optimality across all conceivable optimization contexts, thereby engendering a constraint on its overall applicability. The landscape of ongoing research prospects is rich, and it encompasses the envisagement of binary and multi-objective adaptations of the GOA. Furthermore, the algorithm’s deployment across diverse scientific domains and real-world applications beckons as a fertile avenue for prospective exploration.

## Figures and Tables

**Table 1:** Assigned values to the control parameters of competitor algorithms.

Algorithm	Parameter	Value
GA		
	Type	Real coded.
	Selection	Roulette wheel (Proportionate).
	Crossover	Whole arithmetic (Probability=0.8, $\alpha \in \left[-0.5,1.5\right]$).
	Mutation	Gaussian (Probability=0.05).
PSO		
	Topology	Fully connected.
	Cognitive and social constant	$\left(C_{1},\;C_{2}\right)=\left(2,2\right).$
	Inertia weight	Linear reduction from 0.9 to 0.1
	Velocity limit	10% of the dimension range.
GSA		
	Alpha, $G_{0},R_{norm},R_{power}$	20, 100, 2, 1
TLBO		
	$T_{F}$: the teaching factor	$T_{F}=round\;\left[\left(1+rand\right)\right]$.
	random number *rand*	*rand* is a random number from the interval $\left[0,1\right].$
GWO		
	Convergence parameter (*a*)	a: Linear reduction from 2 to 0.
MVO		
	wormhole existence probability (WEP)	Min(WEP)=0.2 and Max(WEP)=1.
	Exploitation accuracy over the iterations (p)	p=6.
WOA		
	Convergence parameter a	a: Linear reduction from 2 to 0.
	Parameters r and l	r is a random vector in $\left[0,1\right],$
		l is a random number in $\left[-1,1\right].$
TSA		
	$P_{min}$ and $P_{max}$	1, 4
	$c_{1},c_{2},c_{3}$	random numbers lie in the range $\left[0,1\right].$
MPA		
	Constant number	P=0.5,
	Random vector	*R* is a vector of uniform random numbers from $\left[0,1\right].$
	Fish Aggregating Devices (FADs)	FADs=0.2,
	Binary vector	U=0 or 1.
RSA		
	Sensitive parameter α	α=0.1
	Sensitive parameter β	β=0.01
	Evolutionary Sense (ES)	ES are randomly decreasing values between 2 and −2.

**Table 2:** Optimization results of unimodal functions (F1–F7).

		GA	PSO	GSA	TLBO	GWO	MVO	WOA	TSA	MPA	RSA	GOA
F1	mean	34.5437	0.011351	9.92E-17	4.15E-75	8.45E-59	0.141392	6.1E-150	1.26E-47	1.88E-49	6.46E-84	0
	best	21.8428	2.18E-05	5.36E-17	1.46E-76	5.78E-61	0.085887	1.1E-172	1.9E-50	2.3E-52	9.43E-93	0
	worst	48.27454	0.097758	2.23E-16	2.18E-74	6.53E-58	0.229047	1.2E-148	9.02E-47	1.75E-48	1.19E-82	0
	std	8.103596	0.021904	4.27E-17	5.75E-75	1.47E-58	0.035688	2.7E-149	2.43E-47	4.01E-49	2.64E-83	0
	median	33.39374	0.004052	8.44E-17	1.9E-75	4.14E-59	0.134685	2.2E-160	7.09E-49	1.92E-50	3.69E-88	0
	rank	11	9	8	4	5	10	2	7	6	3	1
F2	mean	2.893674	1.469507	5.21E-08	3.96E-39	9.56E-35	0.256823	4.8E-104	1.05E-28	5.4E-28	6.78E-46	0
	best	1.771522	0.129615	3.14E-08	8.03E-40	1.9E-36	0.162297	3.4E-117	1.05E-30	1.58E-29	4.79E-49	0
	worst	4.187515	10.91586	7.28E-08	1.2E-38	2.83E-34	0.393994	9.4E-103	6.38E-28	2.66E-27	5.43E-45	0
	std	0.688131	2.427296	1.18E-08	3.01E-39	8.16E-35	0.061766	2.1E-103	1.6E-28	7.3E-28	1.51E-45	0
	median	2.928265	0.819186	4.9E-08	2.99E-39	7.25E-35	0.25831	8.9E-108	3.99E-29	2.14E-28	3.56E-47	0
	rank	11	10	8	4	5	9	2	6	7	3	1
F3	mean	2151.287	874.6891	474.5464	1.19E-24	6.36E-15	13.69546	19397.34	1.96E-12	2.7E-12	4.76E-58	0
	best	1306.053	38.39523	191.6011	8.36E-29	7.31E-19	6.427129	1155.268	2.74E-17	1.58E-21	1.19E-69	0
	worst	3690.226	5365.03	1028.324	1.56E-23	5.42E-14	23.71885	46521.37	2.32E-11	2.68E-11	5.35E-57	0
	std	651.9863	1532.811	210.8921	3.52E-24	1.38E-14	5.422219	11275.71	5.22E-12	7.22E-12	1.3E-57	0
	median	2013.684	279.0787	413.2552	3.35E-26	1.58E-16	12.17659	22075.51	9.99E-14	1.21E-13	1.49E-61	0
	rank	10	9	8	3	4	7	11	5	6	2	1
F4	mean	3.182379	6.409232	1.347784	4.73E-30	1.34E-14	0.575497	45.73347	0.006311	3.38E-19	1.34E-35	0
	best	2.460207	2.625176	1.93E-08	8.45E-32	7.64E-16	0.203873	0.047089	6.36E-06	3.7E-20	3.83E-40	0
	worst	4.320177	9.826729	3.852453	2.31E-29	1.1E-13	0.98408	88.30133	0.074488	8.65E-19	1.66E-34	0
	std	0.440358	2.122281	1.08719	5.62E-30	2.5E-14	0.180899	32.22163	0.016314	2.17E-19	3.82E-35	0
	median	3.158793	6.156931	1.060589	2.21E-30	4.31E-15	0.59779	42.59176	0.001452	2.94E-19	2.7E-37	0
	rank	9	10	8	3	5	7	11	6	4	2	1
F5	mean	512.3849	4685.587	26.4173	26.85637	26.80292	237.6288	27.19238	28.27683	23.63643	27.45887	0
	best	227.6302	6.358709	25.84867	25.97881	25.29612	26.95008	26.45993	26.48034	22.44977	26.21217	0
	worst	1904.539	90133.63	27.6268	28.55723	27.93213	1709.385	28.5018	28.86228	24.27026	28.59278	0
	std	355.6761	20115.89	0.455677	0.70839	0.739022	398.9057	0.541389	0.706209	0.4456	0.72896	0
	median	440.5026	81.59334	26.32633	26.69676	27.11089	63.20481	27.04987	28.63414	23.65578	27.18532	0
	rank	10	11	3	5	4	9	6	8	2	7	1
F6	mean	34.86707	0.060897	1.25E-16	1.078098	0.705754	0.136757	0.077372	3.848483	1.86E-09	1.54416	0
	best	16.97404	9.5E-06	4.71E-17	0.54459	1.77E-05	0.067418	0.009203	2.589849	1.03E-09	0.862897	0
	worst	73.13031	0.835192	4.43E-16	1.595351	1.724524	0.237401	0.713129	4.796125	4.73E-09	2.393213	0
	std	17.35489	0.184214	9.08E-17	0.298311	0.459725	0.03697	0.153834	0.592668	8.37E-10	0.399298	0
	median	31.4086	0.009074	8.57E-17	1.088354	0.739962	0.14036	0.036663	4.050477	1.71E-09	1.639428	0
	rank	11	4	2	8	7	6	5	10	3	9	1
F7	mean	0.010645	0.164599	0.061373	0.001623	0.000817	0.009972	0.001116	0.005855	0.000615	0.000401	3.56E-05
	best	0.003995	0.095988	0.019791	0.000455	0.00012	0.0056	7.13E-05	0.00218	0.000215	2.99E-05	5.88E-07
	worst	0.017568	0.285254	0.112113	0.003976	0.001565	0.018206	0.003835	0.013816	0.00151	0.000953	0.000105
	std	0.004044	0.052236	0.023729	0.001106	0.000407	0.003234	0.001128	0.003043	0.000314	0.000307	3.34E-05
	median	0.00971	0.153973	0.056558	0.001292	0.000774	0.009512	0.000817	0.004586	0.000571	0.000317	2.71E-05
	rank	9	11	10	6	4	8	5	7	3	2	1
sum rank		71	64	47	33	34	56	42	49	31	28	7
mean rank		10.14286	9.142857	6.714286	4.714286	4.857143	8	6	7	4.428571	4	1
total rank		11	10	7	4	5	9	6	8	3	2	1

**Table 3:** Optimization results of high-dimensional multimodal functions (F8–F13).

		GA	PSO	GSA	TLBO	GWO	MVO	WOA	TSA	MPA	RSA	GOA
F8	mean	−8348.04	−7174.66	−2512.77	−5400.22	−6359.7	−7991.99	−10779.6	−5925.69	−9619.16	−7548.39	−12,569.5
	best	−9571.05	−8778.83	−2969.06	−6984.4	−7834.43	−9028.56	−12,569.5	−7583.05	−10,355.8	−9259.4	−12,569.5
	worst	−6569.32	−5206.21	−2015.67	−4465	−4357.11	−7097.08	−8026.53	−4728.78	−9025.09	−5383.42	−12,569.5
	std	757.8547	895.3058	260.3632	685.2514	877.8481	632.0867	1645.717	716.9043	417.5433	1154.307	1.87E-12
	median	−8603.98	−7160.36	−2498.05	−5345.39	−6523.61	−7972.62	−10,889.5	−5954.46	−9629.9	−7805.26	−12,569.5
	rank	4	7	11	10	8	5	2	9	3	6	1
F9	mean	58.86846	65.44741	29.15228	0	0.980934	103.7514	0	160.3345	0	0	0
	best	16.8933	27.85958	16.9143	0	0	80.65951	0	115.0893	0	0	0
	worst	95.10693	126.4306	46.76302	0	9.129372	141.3836	0	231.9054	0	0	0
	std	19.16566	23.37939	7.881373	0	2.70233	15.81913	0	31.28116	0	0	0
	median	58.96616	62.19288	26.86388	0	0	102.5599	0	165.7714	0	0	0
	rank	4	5	3	1	2	6	1	7	1	1	1
F10	mean	3.546679	2.925435	8.19E−09	4.44E−15	1.58E−14	0.44077	3.2E−15	1.468278	4.09E−15	4.54E−13	8.88E−16
	best	2.698017	1.897756	6.07E−09	4.44E−15	1.15E−14	0.087453	8.88E−16	7.99E−15	8.88E−16	8.88E−16	8.88E−16
	worst	4.12547	4.878091	1.12E−08	4.44E−15	2.22E−14	2.141281	7.99E−15	3.546227	4.44E−15	9.03E−12	8.88E−16
	std	0.430288	0.938045	1.35E−09	0	2.96E−15	0.611269	2.09E−15	1.672925	1.09E−15	2.02E−12	0
	median	3.502708	2.817299	8.05E−09	4.44E−15	1.51E−14	0.133476	4.44E−15	2.22E−14	4.44E−15	8.88E−16	8.88E−16
	rank	11	10	7	4	5	8	2	9	3	6	1
F11	mean	1.582293	0.320849	8.822246	0	0.005007	0.421495	0.009403	0.006064	0	0	0
	best	1.214307	0.006832	3.255758	0	0	0.253372	0	0	0	0	0
	worst	2.154375	2.005863	18.13903	0	0.047681	0.592456	0.081925	0.017241	0	0	0
	std	0.243938	0.466655	4.109461	0	0.011945	0.079737	0.023873	0.006718	0	0	0
	median	1.535265	0.099446	9.042558	0	0	0.423356	0	0.004493	0	0	0
	rank	7	5	8	1	2	6	4	3	1	1	1
F12	mean	0.157037	1.423618	0.127676	0.072387	0.037371	0.960236	0.011252	6.958465	2.31E−10	0.069238	1.62E−32
	best	0.035385	4.47E−05	3.7E−19	0.03343	0.006546	0.000691	0.001059	0.404251	7.31E−11	0.012096	1.57E−32
	worst	0.359563	3.854956	0.635088	0.178688	0.073536	3.859424	0.083424	15.28632	5.8E−10	0.179779	2.54E−32
	std	0.091936	1.221873	0.213301	0.030545	0.019632	0.990186	0.01782	4.73628	1.16E−10	0.039794	2.16E−33
	median	0.147131	1.2003	1.07E−18	0.064612	0.036212	0.641166	0.00666	7.798794	1.93E−10	0.061529	1.57E−32
	rank	8	10	7	6	4	9	3	11	2	5	1
F13	mean	2.614757	3.869959	0.100869	0.983304	0.633228	0.030971	0.261863	2.988147	0.001652	1.803955	7.65E−32
	best	1.149958	0.263545	4.86E−18	0.58094	0.113218	0.016912	0.049589	2.280729	1.28E−09	1.051985	1.35E−32
	worst	4.837813	17.42028	1.222664	1.450667	1.136582	0.066663	0.519796	5.169063	0.010987	2.793816	6.07E−31
	std	1.063186	4.538492	0.28389	0.239798	0.282332	0.012548	0.141363	0.695106	0.004024	0.41072	1.61E−31
	median	2.458018	1.703829	1.46E−17	0.929117	0.638573	0.028099	0.253052	2.728009	3.35E−09	1.694537	1.35E−32
	rank	9	11	4	7	6	3	5	10	2	8	1
sum rank		43	48	40	29	27	37	17	49	12	27	6
mean rank		7.166667	8	6.666667	4.833333	4.5	6.166667	2.833333	8.166667	2	4.5	1
total rank		8	9	7	5	4	6	3	10	2	4	1

**Table 4:** Optimization results of high-dimensional multimodal functions (F14–F23).

		GA	PSO	GSA	TLBO	GWO	MVO	WOA	TSA	MPA	RSA	GOA
F14	mean	1.012829	3.645876	4.086478	1.196416	4.866265	0.998004	2.816492	8.893169	0.998004	4.823742	0.998004
	best	0.998004	0.998004	1.019228	0.998004	0.998004	0.998004	0.998004	0.998004	0.998004	0.998004	0.998004
	worst	1.194013	11.7187	9.831309	2.982105	12.67051	0.998004	10.76318	12.67051	0.998004	12.67051	0.998004
	std	0.045865	3.732496	2.561839	0.610693	4.26894	3.16E−12	2.996068	4.787012	5.09E−17	3.851995	0
	median	0.998006	1.992031	3.970242	0.998004	2.982105	0.998004	1.495018	12.67051	0.998004	3.96825	0.998004
	rank	3	6	7	4	9	2	5	10	1	8	1
F15	mean	0.00603	0.001432	0.002131	0.000453	0.005459	0.004558	0.00063	0.008535	0.000311	0.005053	0.000307
	best	0.000644	0.000307	0.001143	0.00031	0.000307	0.000308	0.000312	0.000308	0.000308	0.000307	0.000307
	worst	0.023479	0.019276	0.004428	0.001241	0.020363	0.020363	0.002178	0.020942	0.000316	0.022553	0.000307
	std	0.007244	0.00422	0.000696	0.0003	0.008834	0.008113	0.000457	0.009974	2.25E−06	0.008991	1.90E−19
	median	0.004113	0.000307	0.00203	0.000315	0.000308	0.000627	0.000481	0.001072	0.000311	0.000653	0.000307
	rank	10	5	6	3	9	7	4	11	2	8	1
F16	mean	−1.03163	−1.03163	−1.03163	−1.03163	−1.03163	−1.03163	−1.03163	−1.03005	−1.03163	−0.99082	−1.03163
	best	−1.03163	−1.03163	−1.03163	−1.03163	−1.03163	−1.03163	−1.03163	−1.03163	−1.03163	−1.03163	−1.03163
	worst	−1.03161	−1.03163	−1.03163	−1.03162	−1.03163	−1.03163	−1.03163	−1	−1.03163	−0.21546	−1.03163
	std	4.59E−06	1.35E−16	1.35E−16	1.59E−06	2.98E−09	3.18E−08	8.38E−11	0.007072	2.28E−16	0.1825	8.31E−17
	median	−1.03163	−1.03163	−1.03163	−1.03163	−1.03163	−1.03163	−1.03163	−1.03163	−1.03163	−1.03163	−1.03163
	rank	7	1	1	6	4	5	3	8	2	9	1
F17	mean	0.422023	0.65439	0.397887	0.403109	0.397898	0.397887	0.397888	0.397909	0.397887	0.397887	0.397887
	best	0.397887	0.397887	0.397887	0.397893	0.397887	0.397887	0.397887	0.397888	0.397887	0.397887	0.397887
	worst	0.832817	1.937365	0.397887	0.500697	0.3981	0.397888	0.39789	0.397946	0.397887	0.397887	0.397887
	std	0.09697	0.526896	0	0.02297	4.74E−05	7.33E−08	7.31E−07	1.7E−05	0	8.97E−16	0
	median	0.398304	0.397887	0.397887	0.397965	0.397888	0.397887	0.397887	0.397907	0.397887	0.397887	0.397887
	rank	8	9	1	7	5	3	4	6	1	2	1
F18	mean	8.421245	3	3	3	7.050008	3	3.000018	14.20181	3	13.8	3
	best	3	3	3	3	3.000001	3	3	3.000001	3	3	3
	worst	30.31682	3	3	3.000002	84.00001	3.000001	3.000147	92.03579	3	84	3
	std	11.11524	2.82E−15	2.79E−15	4.58E−07	18.11215	3.24E−07	3.46E−05	26.59322	1E−15	20.3563	2.88E−16
	median	3.000586	3	3	3	3.000005	3	3.000005	3.000009	3	3	3
	rank	8	2	3	5	7	4	6	10	1	9	1
F19	mean	−3.86265	−3.82413	−3.86278	−3.86051	−3.8621	−3.86278	−3.86068	−3.86225	−3.86278	−3.74604	−3.86278
	best	−3.86278	−3.86278	−3.86278	−3.8627	−3.86278	−3.86278	−3.86276	−3.86278	−3.86278	−3.86278	−3.86278
	worst	−3.86161	−3.08976	−3.86278	−3.85474	−3.8549	−3.86278	−3.8549	−3.85501	−3.86278	−3.08976	−3.86274
	std	0.000338	0.172852	1.92E−15	0.003385	0.002056	2.03E−07	0.002361	0.001753	2.28E−15	0.282864	9.02E−16
	median	−3.86278	−3.86278	−3.86278	−3.86238	−3.86277	−3.86278	−3.86162	−3.86273	−3.86278	−3.86278	−3.86278
	rank	4	9	1	8	6	3	7	5	2	10	1
F20	mean	−3.19843	−3.30089	−3.322	−3.27123	−3.25578	−3.26246	−3.25729	−3.25227	−3.322	−3.19517	−3.322
	best	−3.31774	−3.322	−3.322	−3.31452	−3.32199	−3.32199	−3.32181	−3.32165	−3.322	−3.322	−3.322
	worst	−3.02507	−3.13764	−3.322	−3.15712	−3.13762	−3.20273	−3.08687	−3.08336	−3.322	−1.9217	−3.322
	std	0.081657	0.052988	3.67E−16	0.056572	0.06962	0.06108	0.091694	0.075807	3.81E−16	0.311345	1.41E−17
	median	−3.19362	−3.322	−3.322	−3.30495	−3.26241	−3.26254	−3.32111	−3.26131	−3.322	−3.322	−3.322
	rank	9	3	1	4	7	5	6	8	2	10	1
F21	mean	−4.05229	−6.6523	−6.21031	−6.63717	−9.6474	−8.12535	−9.89304	−5.79327	−10.1532	−8.78928	−10.1532
	best	−7.88766	−10.1532	−10.1532	−9.30299	−10.153	−10.1532	−10.1529	−10.1034	−10.1532	−10.1532	−10.1532
	worst	−2.294	−2.63047	−2.68286	−4.07333	−5.09985	−5.05516	−5.05519	−2.62401	−10.1532	−0.88199	−10.1532
	std	2.072922	3.659234	3.702041	2.127264	1.555137	2.54809	1.138781	3.016364	2.41E−15	3.181731	2.07E−17
	median	−2.62469	−7.62699	−4.18158	−6.69275	−10.1527	−10.1531	−10.1516	−4.95475	−10.1532	−10.1524	−10.1532
	rank	11	7	9	8	4	6	3	10	2	5	1
F22	mean	−6.76101	−8.1775	−9.6989	−8.06962	−10.4024	−8.04873	−8.69025	−7.01929	−10.4029	−8.05397	−10.4029
	best	−10.2388	−10.4029	−10.4029	−9.9566	−10.4029	−10.4029	−10.4029	−10.3661	−10.4029	−10.4029	−10.4029
	worst	−2.5174	−2.75193	−4.67391	−3.94552	−10.402	−2.76589	−2.76539	−2.68875	−10.4029	−0.90808	−10.4029
	std	3.154309	3.169789	1.757215	1.582401	0.000256	3.030069	2.723616	3.582683	3.65E−15	3.599306	1.61E−16
	median	−7.94704	−10.4029	−10.4029	−8.43317	−10.4025	−10.4029	−10.4006	−9.45916	−10.4029	−10.3962	−10.4029
	rank	11	6	4	7	3	9	5	10	2	8	1
F23	mean	−8.18721	−5.6555	−10.5364	−8.03277	−10.536	−9.99793	−9.6541	−7.04655	−10.5364	−7.32853	−10.5364
	best	−10.3471	−10.5364	−10.5364	−9.58103	−10.5363	−10.5364	−10.5362	−10.5028	−10.5364	−10.5364	−10.5364
	worst	−2.66877	−2.42173	−10.5364	−3.95463	−10.5356	−5.12847	−3.83473	−2.41642	−10.5364	−1.85948	−10.5363
	std	2.385851	3.382098	1.58E−15	1.775707	0.000194	1.657273	2.150189	3.807051	2.31E−15	4.034066	1.95E−16
	median	−8.74676	−3.83543	−10.5364	−8.62434	−10.536	−10.5363	−10.5346	−10.1711	−10.5364	−10.508	−10.5364
	rank	6	10	1	7	3	4	5	9	2	8	1
sum rank		77	58	34	59	57	48	48	87	17	77	10
mean rank		7.7	5.8	3.4	5.9	5.7	4.8	4.8	8.7	1.7	7.7	1
total rank		8	6	3	7	5	4	4	9	2	8	1

**Table 5:** Optimization results of the CEC 2017 test suite.

		GOA	RSA	MPA	TSA	WOA	MVO	GWO	TLBO	GSA	PSO	GA
C17-F1	mean	1.00E+02	1.05E+10	3.62E+07	1.78E+09	6.61E+06	7.71E+03	9.05E+07	1.51E+08	7.63E+02	3.22E+03	1.22E+07
	best	1.00E+02	9.05E+09	1.15E+04	3.82E+08	4.82E+06	4.90E+03	2.85E+04	6.72E+07	1.00E+02	3.52E+02	6.29E+06
	worst	1.00E+02	1.25E+10	1.31E+08	3.89E+09	8.71E+06	1.14E+04	3.29E+08	3.64E+08	1.83E+03	9.55E+03	1.74E+07
	std	0.00E+00	1.64E+09	6.77E+07	1.66E+09	1.75E+06	3.21E+03	1.69E+08	1.52E+08	7.96E+02	4.52E+03	4.95E+06
	median	1.00E+02	1.02E+10	6.63E+06	1.43E+09	6.47E+06	7.29E+03	1.66E+07	8.62E+07	5.60E+02	1.50E+03	1.24E+07
	rank	1	11	7	10	5	4	8	9	2	3	6
C17-F3	mean	3.00E+02	9.88E+03	1.44E+03	1.15E+04	1.77E+03	3.00E+02	3.14E+03	7.37E+02	1.05E+04	3.00E+02	1.51E+04
	best	3.00E+02	5.33E+03	8.04E+02	4.37E+03	6.27E+02	3.00E+02	1.56E+03	4.76E+02	6.61E+03	3.00E+02	4.45E+03
	worst	3.00E+02	1.32E+04	2.59E+03	1.62E+04	3.41E+03	3.00E+02	6.03E+03	9.08E+02	1.43E+04	3.00E+02	2.39E+04
	std	0.00E+00	3.84E+03	8.76E+02	5.35E+03	1.39E+03	5.34E-02	2.19E+03	2.01E+02	3.36E+03	0.00E+00	1.08E+04
	median	3.00E+02	1.05E+04	1.17E+03	1.27E+04	1.51E+03	3.00E+02	2.48E+03	7.82E+02	1.06E+04	3.00E+02	1.61E+04
	rank	1	8	5	10	6	3	7	4	9	2	11
C17-F4	mean	4.00E+02	1.38E+03	4.07E+02	5.81E+02	4.26E+02	4.03E+02	4.12E+02	4.09E+02	4.05E+02	4.21E+02	4.15E+02
	best	4.00E+02	8.57E+02	4.03E+02	4.80E+02	4.07E+02	4.02E+02	4.06E+02	4.09E+02	4.04E+02	4.00E+02	4.12E+02
	worst	4.00E+02	1.88E+03	4.12E+02	6.99E+02	4.75E+02	4.05E+02	4.29E+02	4.10E+02	4.06E+02	4.72E+02	4.19E+02
	std	0.00E+00	4.66E+02	4.80E+00	1.14E+02	3.53E+01	1.87E+00	1.21E+01	5.98E-01	1.26E+00	3.67E+01	3.22E+00
	median	4.00E+02	1.38E+03	4.07E+02	5.73E+02	4.11E+02	4.04E+02	4.06E+02	4.10E+02	4.04E+02	4.06E+02	4.15E+02
	rank	1	11	4	10	9	2	6	5	3	8	7
C17-F5	mean	5.01E+02	5.75E+02	5.13E+02	5.67E+02	5.42E+02	5.25E+02	5.13E+02	5.35E+02	5.56E+02	5.29E+02	5.29E+02
	best	5.01E+02	5.60E+02	5.09E+02	5.45E+02	5.24E+02	5.11E+02	5.09E+02	5.30E+02	5.51E+02	5.12E+02	5.24E+02
	worst	5.02E+02	5.91E+02	5.19E+02	6.00E+02	5.80E+02	5.39E+02	5.21E+02	5.39E+02	5.68E+02	5.54E+02	5.35E+02
	std	5.27E-01	1.81E+01	5.59E+00	2.59E+01	2.75E+01	1.27E+01	5.60E+00	4.36E+00	8.74E+00	2.06E+01	5.21E+00
	median	5.01E+02	5.75E+02	5.13E+02	5.61E+02	5.33E+02	5.24E+02	5.12E+02	5.36E+02	5.52E+02	5.25E+02	5.28E+02
	rank	1	11	2	10	8	4	3	7	9	5	6
C17-F6	mean	6.00E+02	6.42E+02	6.01E+02	6.26E+02	6.24E+02	6.02E+02	6.01E+02	6.07E+02	6.18E+02	6.08E+02	6.11E+02
	best	6.00E+02	6.39E+02	6.01E+02	6.16E+02	6.08E+02	6.00E+02	6.01E+02	6.05E+02	6.03E+02	6.01E+02	6.07E+02
	worst	6.00E+02	6.47E+02	6.02E+02	6.42E+02	6.47E+02	6.04E+02	6.02E+02	6.11E+02	6.38E+02	6.20E+02	6.15E+02
	std	0.00E+00	3.71E+00	8.89E-01	1.21E+01	1.75E+01	1.91E+00	5.13E-01	2.71E+00	1.70E+01	8.97E+00	3.72E+00
	median	6.00E+02	6.42E+02	6.01E+02	6.23E+02	6.21E+02	6.02E+02	6.01E+02	6.07E+02	6.15E+02	6.05E+02	6.10E+02
	rank	1	11	3	10	9	4	2	5	8	6	7
C17-F7	mean	7.11E+02	8.08E+02	7.25E+02	8.33E+02	7.64E+02	7.32E+02	7.27E+02	7.54E+02	7.17E+02	7.34E+02	7.38E+02
	best	7.11E+02	7.94E+02	7.21E+02	7.92E+02	7.53E+02	7.17E+02	7.18E+02	7.49E+02	7.15E+02	7.26E+02	7.27E+02
	worst	7.12E+02	8.21E+02	7.30E+02	8.76E+02	7.95E+02	7.52E+02	7.45E+02	7.62E+02	7.21E+02	7.46E+02	7.43E+02
	std	5.44E-01	1.34E+01	4.03E+00	3.91E+01	2.17E+01	1.53E+01	1.32E+01	6.26E+00	2.90E+00	9.45E+00	7.76E+00
	median	7.11E+02	8.08E+02	7.25E+02	8.33E+02	7.55E+02	7.29E+02	7.22E+02	7.52E+02	7.17E+02	7.31E+02	7.41E+02
	rank	1	10	3	11	9	5	4	8	2	6	7
C17-F8	mean	8.01E+02	8.56E+02	8.13E+02	8.50E+02	8.38E+02	8.12E+02	8.16E+02	8.39E+02	8.21E+02	8.24E+02	8.17E+02
	best	8.01E+02	8.44E+02	8.09E+02	8.33E+02	8.19E+02	8.08E+02	8.11E+02	8.32E+02	8.12E+02	8.16E+02	8.13E+02
	worst	8.02E+02	8.61E+02	8.15E+02	8.70E+02	8.50E+02	8.17E+02	8.22E+02	8.48E+02	8.29E+02	8.30E+02	8.26E+02
	std	6.10E-01	8.41E+00	3.03E+00	1.75E+01	1.42E+01	4.18E+00	4.77E+00	8.43E+00	7.35E+00	7.39E+00	5.86E+00
	median	8.01E+02	8.59E+02	8.14E+02	8.49E+02	8.41E+02	8.12E+02	8.17E+02	8.39E+02	8.21E+02	8.24E+02	8.15E+02
	rank	1	11	3	10	8	2	4	9	6	7	5
C17-F9	mean	9.00E+02	1.49E+03	9.05E+02	1.40E+03	1.39E+03	9.01E+02	9.12E+02	9.12E+02	9.00E+02	9.04E+02	9.05E+02
	best	9.00E+02	1.39E+03	9.00E+02	1.18E+03	1.08E+03	9.00E+02	9.01E+02	9.08E+02	9.00E+02	9.01E+02	9.03E+02
	worst	9.00E+02	1.63E+03	9.14E+02	1.70E+03	1.69E+03	9.03E+02	9.34E+02	9.21E+02	9.00E+02	9.13E+02	9.09E+02
	std	0.00E+00	1.10E+02	6.47E+00	2.40E+02	2.71E+02	1.70E+00	1.69E+01	6.20E+00	0.00E+00	6.03E+00	3.14E+00
	median	9.00E+02	1.47E+03	9.04E+02	1.36E+03	1.41E+03	9.00E+02	9.07E+02	9.10E+02	9.00E+02	9.02E+02	9.04E+02
	rank	1	10	5	9	8	2	7	6	1	3	4
C17-F10	mean	1.01E+03	2.63E+03	1.53E+03	2.06E+03	2.06E+03	1.80E+03	1.75E+03	2.21E+03	2.32E+03	1.97E+03	1.74E+03
	best	1.00E+03	2.45E+03	1.40E+03	1.78E+03	1.46E+03	1.47E+03	1.56E+03	1.80E+03	2.03E+03	1.58E+03	1.43E+03
	worst	1.01E+03	3.00E+03	1.61E+03	2.32E+03	2.60E+03	2.32E+03	2.02E+03	2.51E+03	2.43E+03	2.39E+03	2.15E+03
	std	7.07E+00	2.71E+02	1.04E+02	3.05E+02	5.82E+02	4.38E+02	2.11E+02	3.16E+02	2.05E+02	3.56E+02	3.27E+02
	median	1.01E+03	2.53E+03	1.56E+03	2.08E+03	2.08E+03	1.71E+03	1.71E+03	2.26E+03	2.41E+03	1.96E+03	1.69E+03
	rank	1	11	2	8	7	5	4	9	10	6	3
C17-F11	mean	1.10E+03	4.07E+03	1.13E+03	5.59E+03	1.15E+03	1.13E+03	1.16E+03	1.15E+03	1.14E+03	1.14E+03	2.42E+03
	best	1.10E+03	1.47E+03	1.11E+03	5.44E+03	1.11E+03	1.11E+03	1.12E+03	1.14E+03	1.12E+03	1.13E+03	1.12E+03
	worst	1.10E+03	6.64E+03	1.16E+03	5.67E+03	1.18E+03	1.15E+03	1.23E+03	1.17E+03	1.17E+03	1.17E+03	6.13E+03
	std	0.00E+00	2.47E+03	2.35E+01	1.12E+02	3.04E+01	2.37E+01	5.44E+01	1.63E+01	2.29E+01	1.61E+01	2.62E+03
	median	1.10E+03	4.09E+03	1.12E+03	5.62E+03	1.16E+03	1.13E+03	1.14E+03	1.15E+03	1.14E+03	1.14E+03	1.22E+03
	rank	1	10	2	11	7	3	8	6	4	5	9
C17-F12	mean	1.35E+03	7.28E+08	5.86E+05	1.07E+06	2.43E+06	1.06E+06	1.46E+06	5.22E+06	1.05E+06	8.31E+03	6.25E+05
	best	1.32E+03	1.62E+08	2.05E+04	5.57E+05	1.77E+05	9.08E+03	4.69E+04	1.40E+06	4.90E+05	2.56E+03	1.81E+05
	worst	1.44E+03	1.27E+09	9.17E+05	1.32E+06	4.03E+06	3.34E+06	2.29E+06	9.24E+06	1.78E+06	1.43E+04	1.10E+06
	std	6.08E+01	5.97E+08	4.19E+05	3.81E+05	1.90E+06	1.63E+06	1.05E+06	4.41E+06	5.81E+05	5.69E+03	4.02E+05
	median	1.33E+03	7.39E+08	7.03E+05	1.21E+06	2.76E+06	4.52E+05	1.76E+06	5.12E+06	9.71E+05	8.18E+03	6.08E+05
	rank	1	11	3	7	9	6	8	10	5	2	4
C17-F13	mean	1.31E+03	3.55E+07	5.57E+03	1.31E+04	7.78E+03	6.91E+03	1.06E+04	1.72E+04	1.04E+04	6.79E+03	5.62E+04
	best	1.30E+03	2.95E+06	3.80E+03	7.79E+03	3.35E+03	1.39E+03	6.68E+03	1.63E+04	5.17E+03	2.41E+03	8.78E+03
	worst	1.31E+03	1.18E+08	6.82E+03	2.08E+04	1.56E+04	1.27E+04	1.48E+04	1.96E+04	1.46E+04	1.72E+04	1.86E+05
	std	2.41E+00	5.84E+07	1.53E+03	5.96E+03	5.93E+03	6.24E+03	3.54E+03	1.68E+03	4.23E+03	7.46E+03	9.18E+04
	median	1.30E+03	1.06E+07	5.83E+03	1.19E+04	6.09E+03	6.75E+03	1.04E+04	1.65E+04	1.08E+04	3.77E+03	1.50E+04
	rank	1	11	2	8	5	4	7	9	6	3	10
C17-F14	mean	1.40E+03	5.48E+03	1.96E+03	3.45E+03	1.52E+03	1.58E+03	2.38E+03	1.60E+03	5.71E+03	3.05E+03	1.34E+04
	best	1.40E+03	4.79E+03	1.44E+03	1.49E+03	1.48E+03	1.42E+03	1.46E+03	1.52E+03	4.71E+03	1.43E+03	3.81E+03
	worst	1.40E+03	7.08E+03	2.95E+03	5.72E+03	1.56E+03	2.01E+03	5.08E+03	1.63E+03	7.76E+03	7.03E+03	2.67E+04
	std	5.28E-01	1.14E+03	7.56E+02	2.39E+03	4.32E+01	3.09E+02	1.91E+03	5.49E+01	1.52E+03	2.84E+03	1.03E+04
	median	1.40E+03	5.02E+03	1.72E+03	3.30E+03	1.52E+03	1.44E+03	1.48E+03	1.62E+03	5.18E+03	1.87E+03	1.15E+04
	rank	1	9	5	8	2	3	6	4	10	7	11
C17-F15	mean	1.50E+03	1.43E+04	4.06E+03	7.19E+03	6.38E+03	1.54E+03	5.96E+03	1.72E+03	2.46E+04	9.25E+03	4.65E+03
	best	1.50E+03	2.78E+03	3.28E+03	2.35E+03	2.03E+03	1.53E+03	3.64E+03	1.59E+03	1.16E+04	2.92E+03	1.90E+03
	worst	1.50E+03	3.13E+04	5.01E+03	1.29E+04	1.38E+04	1.56E+03	7.08E+03	1.81E+03	3.70E+04	1.52E+04	8.23E+03
	std	2.50E-01	1.32E+04	7.60E+02	4.82E+03	5.47E+03	1.34E+01	1.68E+03	1.16E+02	1.29E+04	5.47E+03	3.34E+03
	median	1.50E+03	1.15E+04	3.97E+03	6.74E+03	4.81E+03	1.55E+03	6.56E+03	1.73E+03	2.50E+04	9.42E+03	4.24E+03
	rank	1	10	4	8	7	2	6	3	11	9	5
C17-F16	mean	1.60E+03	2.03E+03	1.69E+03	2.06E+03	1.96E+03	1.82E+03	1.73E+03	1.68E+03	2.09E+03	1.93E+03	1.81E+03
	best	1.60E+03	1.83E+03	1.64E+03	1.87E+03	1.77E+03	1.73E+03	1.62E+03	1.65E+03	1.96E+03	1.83E+03	1.72E+03
	worst	1.60E+03	2.31E+03	1.72E+03	2.25E+03	2.09E+03	1.89E+03	1.83E+03	1.74E+03	2.29E+03	2.10E+03	1.84E+03
	std	3.35E-01	2.18E+02	3.45E+01	1.84E+02	1.64E+02	7.03E+01	9.49E+01	4.10E+01	1.60E+02	1.33E+02	6.12E+01
	median	1.60E+03	1.99E+03	1.69E+03	2.06E+03	1.99E+03	1.84E+03	1.74E+03	1.66E+03	2.05E+03	1.90E+03	1.84E+03
	rank	1	9	3	10	8	6	4	2	11	7	5
C17-F17	mean	1.70E+03	1.82E+03	1.74E+03	1.81E+03	1.85E+03	1.85E+03	1.77E+03	1.76E+03	1.85E+03	1.75E+03	1.76E+03
	best	1.70E+03	1.80E+03	1.72E+03	1.79E+03	1.78E+03	1.78E+03	1.73E+03	1.75E+03	1.75E+03	1.75E+03	1.75E+03
	worst	1.70E+03	1.83E+03	1.78E+03	1.82E+03	1.90E+03	1.96E+03	1.88E+03	1.77E+03	1.98E+03	1.76E+03	1.76E+03
	std	1.65E-01	1.28E+01	2.87E+01	1.23E+01	5.52E+01	8.94E+01	7.58E+01	1.09E+01	1.26E+02	6.26E+00	2.76E+00
	median	1.70E+03	1.83E+03	1.72E+03	1.81E+03	1.86E+03	1.83E+03	1.74E+03	1.76E+03	1.84E+03	1.75E+03	1.76E+03
	rank	1	8	2	7	9	10	6	5	11	3	4
C17-F18	mean	1.81E+03	5.87E+06	1.13E+04	1.24E+04	2.40E+04	2.15E+04	2.05E+04	3.04E+04	9.96E+03	2.25E+04	1.32E+04
	best	1.80E+03	2.91E+05	4.23E+03	7.64E+03	6.59E+03	8.92E+03	6.46E+03	2.47E+04	6.54E+03	2.91E+03	3.49E+03
	worst	1.82E+03	1.71E+07	1.70E+04	1.67E+04	3.77E+04	3.47E+04	3.46E+04	3.80E+04	1.22E+04	4.19E+04	1.90E+04
	std	1.07E+01	8.24E+06	6.15E+03	4.02E+03	1.59E+04	1.29E+04	1.51E+04	6.50E+03	2.55E+03	2.14E+04	7.19E+03
	median	1.80E+03	3.08E+06	1.21E+04	1.26E+04	2.58E+04	2.13E+04	2.04E+04	2.94E+04	1.06E+04	2.26E+04	1.51E+04
	rank	1	11	3	4	9	7	6	10	2	8	5
C17-F19	mean	1.90E+03	7.26E+05	5.71E+03	1.29E+05	3.58E+04	1.92E+03	5.49E+03	4.78E+03	4.16E+04	2.57E+04	6.32E+03
	best	1.90E+03	4.72E+04	2.33E+03	1.95E+03	7.84E+03	1.91E+03	1.95E+03	2.05E+03	1.14E+04	2.65E+03	2.22E+03
	worst	1.90E+03	1.56E+06	9.65E+03	2.58E+05	6.56E+04	1.93E+03	1.42E+04	1.28E+04	6.04E+04	7.92E+04	1.01E+04
	std	7.90E-01	7.24E+05	3.96E+03	1.56E+05	2.52E+04	7.69E+00	6.21E+03	5.69E+03	2.33E+04	3.83E+04	3.46E+03
	median	1.90E+03	6.48E+05	5.44E+03	1.29E+05	3.49E+04	1.91E+03	2.92E+03	2.13E+03	4.73E+04	1.04E+04	6.45E+03
	rank	1	11	5	10	8	2	4	3	9	7	6
C17-F20	mean	2.00E+03	2.23E+03	2.10E+03	2.21E+03	2.21E+03	2.14E+03	2.18E+03	2.07E+03	2.26E+03	2.17E+03	2.05E+03
	best	2.00E+03	2.17E+03	2.07E+03	2.11E+03	2.10E+03	2.05E+03	2.13E+03	2.06E+03	2.19E+03	2.15E+03	2.04E+03
	worst	2.00E+03	2.29E+03	2.13E+03	2.33E+03	2.30E+03	2.26E+03	2.25E+03	2.08E+03	2.36E+03	2.21E+03	2.06E+03
	std	0.00E+00	6.14E+01	2.35E+01	9.93E+01	9.92E+01	9.01E+01	5.68E+01	9.84E+00	8.47E+01	3.04E+01	1.12E+01
	median	2.00E+03	2.23E+03	2.09E+03	2.21E+03	2.23E+03	2.14E+03	2.16E+03	2.07E+03	2.25E+03	2.17E+03	2.06E+03
	rank	1	10	4	9	8	5	7	3	11	6	2
C17-F21	mean	2.20E+03	2.27E+03	2.26E+03	2.33E+03	2.31E+03	2.25E+03	2.32E+03	2.30E+03	2.37E+03	2.32E+03	2.30E+03
	best	2.20E+03	2.22E+03	2.26E+03	2.22E+03	2.22E+03	2.20E+03	2.31E+03	2.20E+03	2.36E+03	2.31E+03	2.23E+03
	worst	2.20E+03	2.29E+03	2.26E+03	2.38E+03	2.36E+03	2.31E+03	2.32E+03	2.34E+03	2.39E+03	2.33E+03	2.34E+03
	std	0.00E+00	3.28E+01	2.33E+00	7.72E+01	6.76E+01	6.72E+01	4.13E+00	7.06E+01	1.59E+01	8.41E+00	5.30E+01
	median	2.20E+03	2.28E+03	2.26E+03	2.36E+03	2.34E+03	2.25E+03	2.32E+03	2.33E+03	2.37E+03	2.32E+03	2.32E+03
	rank	1	4	3	10	7	2	8	6	11	9	5
C17-F22	mean	2.30E+03	2.94E+03	2.31E+03	2.73E+03	2.32E+03	2.29E+03	2.31E+03	2.32E+03	2.30E+03	2.31E+03	2.32E+03
	best	2.30E+03	2.72E+03	2.30E+03	2.45E+03	2.32E+03	2.23E+03	2.30E+03	2.31E+03	2.30E+03	2.30E+03	2.32E+03
	worst	2.30E+03	3.09E+03	2.31E+03	2.94E+03	2.33E+03	2.31E+03	2.32E+03	2.33E+03	2.30E+03	2.35E+03	2.32E+03
	std	1.54E-01	1.67E+02	3.89E+00	2.31E+02	6.03E+00	4.12E+01	1.07E+01	9.03E+00	5.92E-03	2.36E+01	3.45E+00
	median	2.30E+03	2.96E+03	2.31E+03	2.76E+03	2.32E+03	2.30E+03	2.31E+03	2.32E+03	2.30E+03	2.30E+03	2.32E+03
	rank	3	11	4	10	9	1	5	8	2	6	7
C17-F23	mean	2.60E+03	2.70E+03	2.61E+03	2.73E+03	2.65E+03	2.62E+03	2.61E+03	2.64E+03	2.80E+03	2.65E+03	2.66E+03
	best	2.60E+03	2.67E+03	2.61E+03	2.64E+03	2.63E+03	2.61E+03	2.61E+03	2.63E+03	2.73E+03	2.64E+03	2.64E+03
	worst	2.60E+03	2.75E+03	2.62E+03	2.77E+03	2.67E+03	2.63E+03	2.62E+03	2.65E+03	2.94E+03	2.66E+03	2.67E+03
	std	1.40E+00	3.58E+01	2.70E+00	6.62E+01	2.25E+01	1.18E+01	7.18E+00	9.79E+00	1.05E+02	9.53E+00	1.48E+01
	median	2.60E+03	2.70E+03	2.61E+03	2.75E+03	2.65E+03	2.62E+03	2.61E+03	2.64E+03	2.76E+03	2.64E+03	2.66E+03
	rank	1	9	3	10	7	4	2	5	11	6	8
C17-F24	mean	2.63E+03	2.86E+03	2.63E+03	2.67E+03	2.77E+03	2.69E+03	2.75E+03	2.76E+03	2.75E+03	2.77E+03	2.73E+03
	best	2.52E+03	2.83E+03	2.62E+03	2.52E+03	2.74E+03	2.50E+03	2.73E+03	2.75E+03	2.50E+03	2.76E+03	2.53E+03
	worst	2.73E+03	2.92E+03	2.64E+03	2.81E+03	2.79E+03	2.76E+03	2.77E+03	2.77E+03	2.90E+03	2.79E+03	2.81E+03
	std	1.24E+02	4.34E+01	7.70E+00	1.68E+02	2.28E+01	1.31E+02	1.84E+01	7.73E+00	1.84E+02	1.43E+01	1.39E+02
	median	2.64E+03	2.84E+03	2.63E+03	2.67E+03	2.76E+03	2.74E+03	2.75E+03	2.76E+03	2.80E+03	2.77E+03	2.78E+03
	rank	1	11	2	3	9	4	7	8	6	10	5
C17-F25	mean	2.93E+03	3.29E+03	2.92E+03	3.14E+03	2.91E+03	2.92E+03	2.94E+03	2.93E+03	2.92E+03	2.92E+03	2.95E+03
	best	2.90E+03	3.22E+03	2.91E+03	2.90E+03	2.76E+03	2.90E+03	2.92E+03	2.92E+03	2.90E+03	2.90E+03	2.94E+03
	worst	2.95E+03	3.37E+03	2.92E+03	3.68E+03	2.96E+03	2.94E+03	2.95E+03	2.95E+03	2.94E+03	2.95E+03	2.96E+03
	std	2.45E+01	6.63E+01	4.38E+00	3.88E+02	1.05E+02	2.71E+01	1.31E+01	2.16E+01	2.53E+01	2.83E+01	1.08E+01
	median	2.94E+03	3.28E+03	2.92E+03	2.99E+03	2.95E+03	2.92E+03	2.94E+03	2.93E+03	2.92E+03	2.92E+03	2.95E+03
	rank	6	11	2	10	1	3	8	7	4	5	9
C17-F26	mean	2.90E+03	3.79E+03	3.02E+03	3.65E+03	3.19E+03	2.90E+03	3.28E+03	3.22E+03	3.89E+03	2.90E+03	2.90E+03
	best	2.90E+03	3.45E+03	2.89E+03	3.15E+03	2.93E+03	2.90E+03	2.97E+03	2.91E+03	2.80E+03	2.80E+03	2.70E+03
	worst	2.90E+03	4.13E+03	3.31E+03	4.32E+03	3.62E+03	2.90E+03	3.94E+03	3.91E+03	4.40E+03	3.01E+03	3.12E+03
	std	3.94E-13	3.13E+02	2.07E+02	6.04E+02	3.20E+02	3.93E-02	4.74E+02	4.93E+02	7.84E+02	9.08E+01	2.24E+02
	median	2.90E+03	3.78E+03	2.93E+03	3.56E+03	3.11E+03	2.90E+03	3.10E+03	3.02E+03	4.19E+03	2.90E+03	2.89E+03
	rank	2	10	5	9	6	3	8	7	11	4	1
C17-F27	mean	3.09E+03	3.24E+03	3.11E+03	3.18E+03	3.20E+03	3.09E+03	3.12E+03	3.12E+03	3.23E+03	3.14E+03	3.16E+03
	best	3.09E+03	3.13E+03	3.09E+03	3.10E+03	3.18E+03	3.09E+03	3.09E+03	3.10E+03	3.22E+03	3.10E+03	3.12E+03
	worst	3.09E+03	3.43E+03	3.14E+03	3.23E+03	3.21E+03	3.10E+03	3.18E+03	3.17E+03	3.25E+03	3.19E+03	3.22E+03
	std	2.79E-13	1.44E+02	2.15E+01	5.94E+01	1.27E+01	2.71E+00	4.44E+01	4.11E+01	1.65E+01	3.98E+01	4.62E+01
	median	3.09E+03	3.19E+03	3.10E+03	3.20E+03	3.20E+03	3.09E+03	3.10E+03	3.10E+03	3.23E+03	3.13E+03	3.15E+03
	rank	1	11	3	8	9	2	5	4	10	6	7
C17-F28	mean	3.10E+03	3.80E+03	3.22E+03	3.60E+03	3.29E+03	3.24E+03	3.35E+03	3.33E+03	3.46E+03	3.31E+03	3.25E+03
	best	3.10E+03	3.72E+03	3.17E+03	3.42E+03	3.15E+03	3.10E+03	3.20E+03	3.22E+03	3.45E+03	3.18E+03	3.15E+03
	worst	3.10E+03	3.86E+03	3.25E+03	3.82E+03	3.40E+03	3.40E+03	3.42E+03	3.40E+03	3.48E+03	3.40E+03	3.53E+03
	std	0.00E+00	7.21E+01	3.88E+01	2.18E+02	1.34E+02	1.76E+02	1.11E+02	9.24E+01	1.61E+01	1.06E+02	1.96E+02
	median	3.10E+03	3.81E+03	3.24E+03	3.58E+03	3.31E+03	3.24E+03	3.40E+03	3.36E+03	3.46E+03	3.33E+03	3.17E+03
	rank	1	11	2	10	5	3	8	7	9	6	4
C17-F29	mean	3.13E+03	3.38E+03	3.21E+03	3.24E+03	3.36E+03	3.21E+03	3.27E+03	3.22E+03	3.35E+03	3.27E+03	3.24E+03
	best	3.13E+03	3.31E+03	3.17E+03	3.17E+03	3.24E+03	3.14E+03	3.19E+03	3.17E+03	3.24E+03	3.17E+03	3.19E+03
	worst	3.13E+03	3.45E+03	3.25E+03	3.31E+03	3.51E+03	3.29E+03	3.39E+03	3.24E+03	3.65E+03	3.36E+03	3.29E+03
	std	2.64E+00	7.84E+01	3.79E+01	6.30E+01	1.20E+02	6.70E+01	9.89E+01	3.58E+01	2.12E+02	9.02E+01	4.52E+01
	median	3.13E+03	3.39E+03	3.20E+03	3.24E+03	3.34E+03	3.19E+03	3.25E+03	3.23E+03	3.26E+03	3.28E+03	3.24E+03
	rank	1	11	3	5	10	2	7	4	9	8	6
C17-F30	mean	3.42E+03	3.78E+06	4.27E+05	6.33E+05	1.02E+06	3.12E+05	9.63E+05	6.23E+04	8.06E+05	3.99E+05	1.57E+06
	best	3.39E+03	8.52E+05	1.63E+04	1.16E+05	4.50E+03	7.56E+03	3.45E+04	3.01E+04	6.19E+05	6.48E+03	5.41E+05
	worst	3.44E+03	5.98E+06	6.30E+05	1.34E+06	3.86E+06	1.19E+06	1.39E+06	1.05E+05	1.03E+06	7.90E+05	3.58E+06
	std	2.95E+01	2.28E+06	2.96E+05	5.51E+05	2.01E+06	6.21E+05	6.78E+05	3.87E+04	1.81E+05	4.80E+05	1.52E+06
	median	3.42E+03	4.15E+06	5.31E+05	5.39E+05	1.12E+05	2.53E+04	1.21E+06	5.73E+04	7.87E+05	3.99E+05	1.08E+06
	rank	1	11	5	6	9	3	8	2	7	4	10
Sum rank		37	294	99	251	213	106	173	175	210	167	179
Mean rank		1.28E+00	1.01E+01	3.41E+00	8.66E+00	7.34E+00	3.66E+00	5.97E+00	6.03E+00	7.24E+00	5.76E+00	6.17E+00
Total rank		1	11	2	10	9	3	5	6	8	4	7

**Table 6:** Evaluation results of real-world applications.

DP		GOA	RSA	MPA	TSA	WOA	MVO	GWO	TLBO	GSA	PSO	GA
TCS	mean	0.012602	0.013292	0.012677	0.012992	0.01332	0.016708	0.012738	0.018401	0.019829	2.21E+13	1.73E+12
	best	0.012602	0.013206	0.012677	0.012695	0.012683	0.012768	0.012683	0.017838	0.013115	0.017729	0.01826
	worst	0.012602	0.013444	0.012677	0.013588	0.014618	0.018229	0.012975	0.019043	0.033208	3.93E+14	1.79E+13
	std	7.63E-18	8.14E-05	3.34E-09	0.000283	0.000709	0.001933	6.49E-05	0.00042	0.004999	9.75E+13	5.73E+12
	median	0.012602	0.01327	0.012677	0.012914	0.01311	0.017677	0.012736	0.018354	0.019378	0.017729	0.026318
	rank	1	5	2	4	6	7	3	8	9	11	10
WB	mean	1.72468	2.216423	1.726575	1.746197	2.354864	1.744131	1.729149	3.57E+13	2.497481	4.92E+13	1.21E+13
	best	1.72468	1.995372	1.726575	1.73622	1.830046	1.730342	1.727295	3.119519	2.112272	4.191127	2.837774
	worst	1.72468	2.589229	1.726575	1.756055	4.215966	1.780406	1.733486	3.44E+14	2.828401	2.98E+14	1.31E+14
	std	2.53E-16	0.171437	3.99E-09	0.006667	0.76323	0.016362	0.001621	9.65E+13	0.227781	1.04E+14	4.11E+13
	median	1.72468	2.189572	1.726575	1.7463	2.113632	1.739766	1.728886	5.980438	2.529163	7.094138	5.944616
	rank	1	6	2	5	7	4	3	10	8	11	9
SR	mean	2996.348	3300.259	2999.345	3037.742	3164.274	3035.264	3008.222	7.46E+13	3491.113	1.1E+14	5.3E+13
	best	2996.348	3199.444	2999.345	3018.152	3044.306	3012.098	3004.887	5439.3	3185.38	3327.892	3375.447
	worst	2996.348	3362.826	2999.345	3052.476	3480.881	3078.566	3014.622	5.4E+14	4157.493	5.58E+14	3.42E+14
	std	1.03E-12	68.44571	3.79E-06	12.06662	126.4984	15.77681	2.983754	1.38E+14	312.033	1.48E+14	9.26E+13
	median	2996.348	3316.223	2999.345	3039.661	3128.502	3035.736	3007.666	2.92E+13	3351.996	7.88E+13	2.12E+13
	rank	1	7	2	5	6	4	3	10	8	11	9
PV	mean	5882.895	13,838.04	5888.784	6361.634	8465.669	6662.434	6046.469	33,159.47	23,866.74	34,881.96	29,693.68
	best	5882.895	8174.803	5888.784	5921.028	6361.305	6036.095	5897.686	11,886.59	13,320.2	10,901.6	12,024.12
	worst	5882.895	23,072.84	5888.784	7186.495	14,320.11	7310.76	6848.651	72,180.9	37,826.84	60,489.28	54,178.55
	std	2.07E-12	4223.96	4.97E-06	450.0219	2271.864	432.6787	323.3558	18,634.81	9065.95	17,445.68	14,627.29
	median	5882.895	12,612.46	5888.784	6206.324	7955.339	6728.311	5907.843	29,142.83	22,876.3	38,562.36	26,190.2
	rank	1	7	2	4	6	5	3	10	8	11	9
Sum rank		4	25	8	18	25	20	12	38	33	44	37
Mean rank		1	6.25	2	4.5	6.25	5	3	9.5	8.25	11	9.25
Total ranking		1	6	2	4	6	5	3	9	7	10	8

**Table 7:** Wilcoxon rank sum test results.

Compared Algorithm	Objective Function Type				
	F1 to F7	F8 to F13	F14 to F23	CEC 2017	Engineering Problems
GOA vs. RSA	3.6E-21	1.97E-21	1.97E-21	1.97E-21	2.79E-07
GOA vs. MPA	2.43E-05	4.35E-10	1.49E-11	3.34E-15	6.24E-06
GOA vs. TSA	7.23E-21	1.97E-21	1.97E-21	1.97E-21	1.37E-07
GOA vs. WOA	9.04E-21	1.97E-21	1.97E-21	1.97E-21	1.84E-07
GOA vs. MVO	8.04E-17	5.13E-19	5.68E-20	6.41E-20	4.64E-07
GOA vs. GWO	1.44E-18	9.98E-21	5.64E-21	9.5E-21	1.49E-06
GOA vs. TLBO	7.06E-20	1.97E-21	1.97E-21	1.97E-21	2.28E-07
GOA vs. GSA	3.02E-20	2.87E-21	1.97E-21	1.97E-21	3.57E-08
GOA vs. PSO	6.25E-20	9.98E-21	1.72E-20	2.24E-21	7.06E-07
GOA vs. GA	3.09E-20	1.97E-21	2.02E-21	1.97E-21	1.59E-07

**Table 8:** Results for GOA and competitor algorithms in EC problem (normal mode).

Algorithm	Mean (Dollar)	Best (Dollar)	Worst (Dollar)	Std (Dollar)	Median (Dollar)	Rank
GOA	2.1148E07	2.1056E07	2.1297E07	7.5142E01	2.1137E07	1
GA	8.5146E08	8.4236E08	8.5891E08	2.6145E06	8.4809E08	11
PSO	5.2158E08	5.0369E08	5.2621E08	1.2485E06	5.1262E08	10
GSA	6.7624E07	6.4512E07	6.8002E07	5.2176E04	6.4849E07	9
TLBO	3.2648E07	3.0914E07	3.2861E07	7.5423E03	3.1562E07	8
GWO	2.7592E07	2.5627E07	2.7806E07	8.6427E02	2.6208E07	7
MVO	2.4257E07	2.1045E07	2.4614E07	6.5654E02	2.2162E07	6
WOA	2.1739E07	2.1296E07	2.2146E07	2.7865E02	2.1428E07	3
MPA	2.2365E07	2.1624E07	2.2948E07	1.4552E02	2.2061E07	5
TSA	2.1562E07	2.1204E07	2.1854E07	1.6254E02	2.1454E07	2
RSA	2.1851E07	2.1425E07	2.2004E07	1.1986E02	2.1619E07	4

**Table 9:** Results for GOA and competitor algorithms in EC problem (abnormal mode).

Algorithm	Mean (Dollar)	Best (Dollar)	Worst (Dollar)	Std (Dollar)	Median (Dollar)	Rank
GOA	2.34E+07	2.28E+07	2.55E+07	8.24E+01	2.29E+07	1
GA	9.79E+08	9.48E+08	9.94E+08	2.94E+06	9.60E+08	11
PSO	6.41E+08	6.10E+08	6.58E+08	1.34E+06	6.31E+08	10
GSA	8.59E+07	8.49E+07	8.71E+07	5.89E+04	8.55E+07	9
TLBO	4.27E+07	4.10E+07	4.48E+07	7.99E+03	4.25E+07	8
GWO	3.24E+07	3.00E+07	3.28E+07	9.37E+02	3.19E+07	7
MVO	3.21E+07	3.05E+07	3.34E+07	6.96E+02	3.18E+07	6
WOA	2.78E+07	2.73E+07	2.84E+07	2.97E+02	2.76E+07	5
MPA	2.66E+07	2.59E+07	2.69E+07	1.66E+02	2.64E+07	3
TSA	2.68E+07	2.64E+07	2.70E+07	1.83E+02	2.65E+07	4
RSA	2.46E+07	2.37E+07	2.58E+07	1.30E+02	2.40E+07	2

## Data Availability

Not applicable.
